# Ocean dominated expansion and contraction of the late Quaternary tropical rainbelt

**DOI:** 10.1038/s41598-017-09816-8

**Published:** 2017-08-24

**Authors:** Joy S. Singarayer, Paul J. Valdes, William H. G. Roberts

**Affiliations:** 1 Department of Meteorology and Centre for Past Climate Change, University of Reading, Reading, United Kingdom; 2 Bristol Research Initiative for the Dynamic Global Environment (BRIDGE), School of Geographical Sciences, University of Bristol, University Road, Bristol, United Kingdom

## Abstract

The latitude of the tropical rainbelt oscillates seasonally but has also varied on millennial time-scales in response to changes in the seasonal distribution of insolation due to Earth’s orbital configuration, as well as climate change initiated at high latitudes. Interpretations of palaeoclimate proxy archives often suggest hemispherically coherent variations, some proposing meridional shifts in global rainbelt position and the ‘global monsoon’, while others propose interhemispherically symmetric expansion and contraction. Here, we use a unique set of climate model simulations of the last glacial cycle (120 kyr), that compares well against a compilation of precipitation proxy data, to demonstrate that while asymmetric extratropical forcings (icesheets, freshwater hosing) generally produce meridional shifts in the zonal mean rainbelt, orbital variations produce expansion/contractions in terms of the global zonal mean. This is primarily a dynamic response of the rainbelt over the oceans to regional interhemispheric temperature gradients, which is opposite to the largely local thermodynamic terrestrial response to insolation. The mode of rainbelt variation is regionally variable, depending on surface type (land or ocean) and surrounding continental configuration. This makes interpretation of precipitation-proxy records as large-scale rainbelt movement challenging, requiring regional or global data syntheses.

## Introduction

The Inter-Tropical Convergence Zone (ITCZ) describes the band of low-level convergence, convective activity, and rainfall due to radiative heating in equatorial regions. The ITCZ and its associated rainbelt seasonally move north and south as the latitude of maximum solar insolation varies. Future changes in the position and intensity of the tropical rainbelt are projected in response to global warming^1^. Predicting the nature of changes in the rainbelt is of critical importance for agriculture and water security in low-latitude regions^2^, yet currently there is a high degree of uncertainty in model projections of tropical hydroclimatic change^1^.

Over the Quaternary there has been much variation in the position of the rainbelt. The solstices precess around the Earth’s eccentric orbit with a periodicity of ~21 kyr, altering the seasonal distribution of insolation. When perihelion occurs in northern hemisphere summer (resulting in seasonal higher insolation) proxy records indicate enhanced precipitation in the northern subtropics^3, 4^, ascribed to intensified monsoon circulation resulting from greater land-sea temperature contrasts. This advects more moisture to the continents and is often interpreted as a northward movement of the rainbelt^3–5^. Model simulations have also demonstrated a response of precipitation^6^ and ITCZ^6, 7^ to insolation seasonality. Precipitation-proxy reconstructions^3, 8^ have suggested that the northern and southern hemispheres have operated in anti-phase on multi-millennial time-scales. When summer insolation, and therefore precipitation, is strongest in one hemisphere it is weakest in the other. By extension, these studies have inferred that the seasonal extremes of the rainbelt respond to local summer insolation, and consequently the rainbelt seasonal range undergoes latitudinal migrations^3, 5, 9^.

There is also a high degree of correlation between tropical precipitation proxies and Greenland ice-core δ^18^O^3, 10, 11^, suggesting meridional shifts of the rainbelt occurred in response to extra-tropical forcing on millennial time-scales (e.g. Dansgaard-Oschger events). Models indicate that the rainbelt migrates southwards in response to altered interhemispheric temperature gradients^12^ or to scavenge heat from the warmer southern hemisphere^13^, although the physical mechanism is debated^12–15^. Conversely, there is evidence suggestive of hemispherically symmetric expansion and contraction of the rainbelt in response to glacial cycle drivers (eg. CO_2_^16^, and Heinrich events^17, 18^). These diverse interpretations have led to considerable debate in the literature concerning the mode and mechanisms of variation of the rainbelt^5, 8, 18, 19^. Model-based studies have often focussed on the global response of the central location of the rainbelt^7, 20^, or are limited to few time periods^7, 21^, making it difficult to assess spatiotemporal patterns of rainbelt response.

Here, we address the questions of how the global tropical rainbelt responds to multi-millennial forcing during a complete glacial cycle and how this is expressed regionally. Using the Hadley Centre coupled ocean-atmosphere-vegetation model^22–24^ we simulated time slices covering the last 120 kyr. Four sets of sensitivity experiments were performed: **ORB−ONLY**, where the orbital configuration varies with each time slice; **ORB+GHG**, varying orbit and atmospheric greenhouse gases; **ALL**, where ice-sheets and sea-level vary in addition; and **HE**, where freshwater was added to the North Atlantic to simulate Heinrich events (see Methods).

## Results

### Global patterns

**Figure 1:**
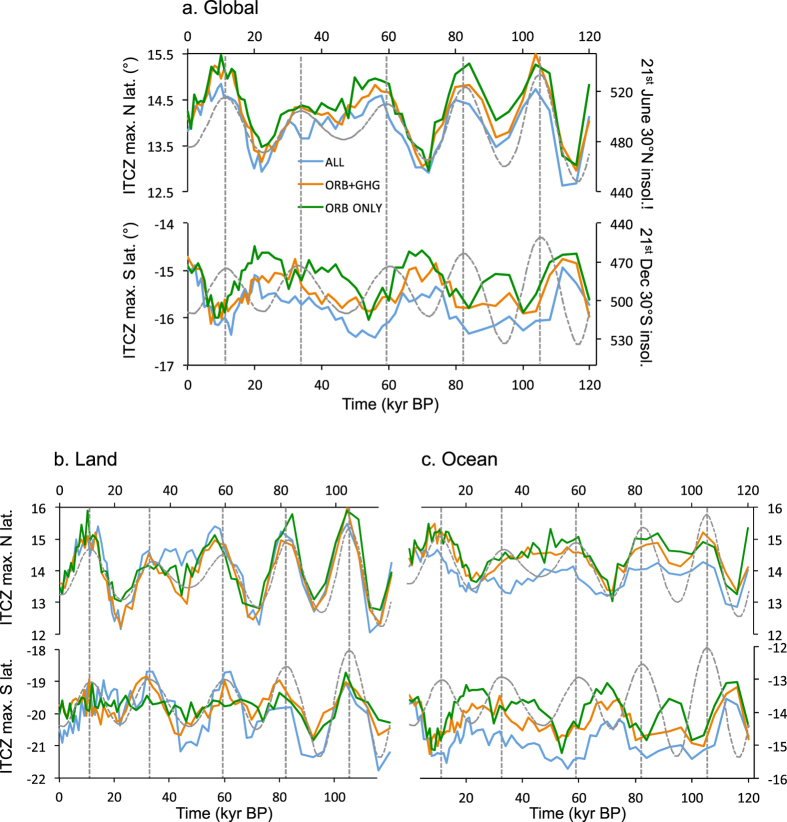
HadCM3 modelled variation in the global ITCZ position during the last glacial cycle. (**a**) Top: northern hemisphere maximum limit of the global mean tropical rainbelt (ITCZ) in any month for the ORB−ONLY (green), ORB+GHG (orange), and ALL (blue) set of simulations. Boreal summer insolation is plotted for comparison in grey dotted lines and shows a high level of similarity to the rainbelt position. Bottom: southern hemisphere maximum limit of the tropical rainbelt in any month, and austral summer insolation (grey dotted lines), demonstrating that the southern limit is roughly anti-phased with local insolation. (**b**) Same as in (**a**) but only for land points, excluding coastal grid cells. (**c**) Same as in (**a**) but only averaged over marine data points.

The extreme northern latitude of the model global mean rainbelt varies in phase with boreal summer insolation (Fig. 1a, top), in that a lag-correlation analysis produces the highest correlation coefficients with 0° lag. However, the southern hemisphere summer extreme rainbelt latitude varies in anti-phase with austral summer insolation (highest correlation obtained at 180° with 21^st^ December 30°S insolation; Fig. 1a, bottom). In other words, in response to changes in orbital configuration the latitudinal range covered by the rainbelt as it moves through its seasonal cycle contracts and expands rather than undergoing a wholesale latitudinal shift. This is contrary to expectations if local insolation is the key factor in each hemisphere, as argued by some studies^3, 4, 9^. Comparison of the ORB, ORB+GHG, and ALL experiments (Fig. 1) to examine the impacts of the different driving forces shows that the expansion of ice-sheets, occurring predominantly in the northern hemisphere, induces a southward movement of both northern and southern extremes, i.e. a latitudinal shift of the mean rainbelt, in contrast to the seasonal insolation response. The response to decreases in CO_2_ (ORG+GHG) is also generally a southward shift in the mean rainbelt compared to ORB−ONLY.

**Figure 2:**
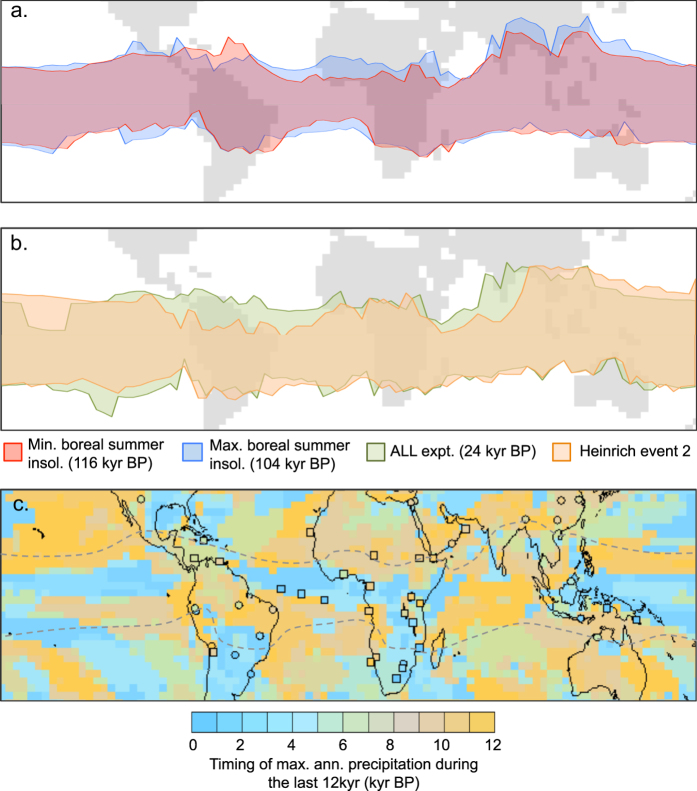
Seasonal range of the experiment ALL tropical rainbelt under different boundary conditions, and comparison of the model simulations with relevant palaeodata. (**a**) Comparison of boreal minimum summer insolation time slice (116 kyr in red) and boreal maximum summer insolation time slice (104 kyr in blue). The same plot is shown in Supplementary Information Fig. S17 with uncertainties included as error bars based on the standard deviation of different averaging periods to account for interannual variability; (**b**) Comparison of simulated Heinrich event (HE2 in orange) rainbelt seasonal range with the equivalent control simulation from the ALL experiment (24 kyr in green); (**c**) map of the time of maximum precipitation over the last 12 kyr from each modelled grid point in the ALL simulation, and symbols representing the equivalent timing from compiled palaeoprecipitation proxy records. The circle symbols represent inferences made from water isotope records (δ^18^O or δD) and squares represent other precipitation proxy sources. See Supplementary Information Table 1 for details of the individual records. Figure maps created using Ferret software (http://ferret.pmel.noaa.gov/Ferret/home) for Linux 64-bit.

The mapped rainbelt seasonal ranges during times of boreal summer perihelion (Fig. 2a, blue) and aphelion (Fig. 2a, red) show that oceanic regions dominate the contraction/expansion of the modelled global mean response (Figs 1c and 2a); particularly visible in the Atlantic and East Pacific. Terrestrial regions, however, generally demonstrate meridional shifts (Figs 1b and 2a), displayed in many of the palaeorecords^3, 5, 9^, where the northern and southern limits of the mean terrestrial rainbelt correlate with local insolation. The contrasting pattern of the marine and terrestrial rainbelts is similarly demonstrated by empirical orthogonal function analysis (Figs S1, S2). The opposing land/ocean response to seasonal insolation differs markedly from the response to northern extratropical freshwater hosing (HE), which produces a southward shift (Fig. 2b) in the rainbelt over most oceanic and terrestrial regions (Supplementary Information section 1), except West Pacific/East Asia.

We mapped (Fig. 2c) the timing of maximum annual mean precipitation over the last 12 kyr to enable comparison of the model with a compilation of palaeo-precip proxy data (Table S1). The local timing of maximum annual mean precipitation in the model in general matches the timing of rainy season maximum precipitation, and therefore is an indicator of the intensity of the rainbelt in the season it passes over each location. A difference in timing of maximum annual precipitation of ~10 kyr between northern and southern maximum extension would be observed if the rainbelt undergoes latitudinal migrations, but close to zero for expansion/contraction. The model and data both show considerable regional complexity in the timing of maximum precipitation, but there is a generally favourable comparison of modelled patterns with synthesised regional palaeorecords (Fig. 2c). The fidelity of the modelled precipitation variations has been demonstrated over Africa previously^25^ and for other regions in Supplementary Information section 2.1 and Fig. S3. A clear north/south pattern can be seen over the large landmasses that span the equator in that northern terrestrial maximum precipitation generally occurs in the early Holocene (yellow colours) whereas southern terrestrial maximum occurs in the late Holocene/present (blue colours). Thus there is a difference of 8–12 kyr between north and south (Fig. 2c), indicative of a latitudinal shift in rainbelt precipitation.

### Oceanic variations

Over marine regions, particularly the Atlantic and eastern Pacific, the expansion/contraction mode of variation is expressed as maximum precipitation in the early Holocene (yellow colours; Fig. 2c) to the north and south of the equator, and maximum in the late Holocene/pre-industrial over the equator itself (blues; Fig. 2c). This expansion/contraction is the result of the different response of the marine ITCZ when at its northern and southern extremes. Both responses, however, are caused by changes in the interhemispheric temperature gradient. In boreal summer, when the ITCZ is farthest north, if the insolation is higher, the ITCZ is drawn farther away from the equator by the increased heating in the northern hemisphere.

**Figure 3:**
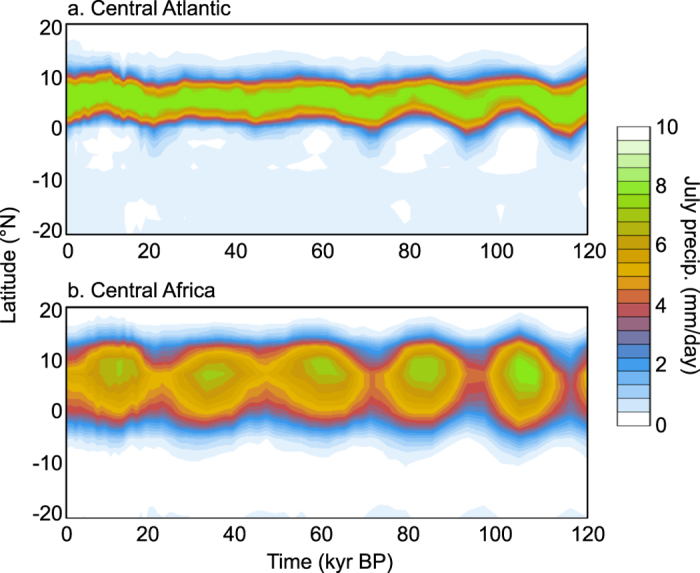
Time evolution of the July mean precipitation averaged over (**a**) the Central Atlantic, 330–345°E average, and (**b**) Central Africa (15–30°E) in the ORB-ONLY experiment. A comparison of the oceanic and terrestrial regions highlights the difference in seasonal response to changes in insolation distribution, whereby the oceanic rainbelt moves north/south and the terrestrial rainbelt responds more thermodynamically.

Conversely, when boreal summer insolation is lower it moves towards the equator (Fig. 3a). In boreal winter, when the ITCZ is farthest south, if the insolation is lower, it is lower in both the northern and southern hemispheres and, although the southern hemisphere cools, because of the greater proportion of land in the northern hemisphere, there is actually greater cooling in the northern hemisphere (Fig. S6). Consequently, the boreal winter rainbelt is located further south in response to the increased interhemispheric temperature gradient. Conversely, when austral summer (boreal winter) insolation is high the rainbelt is located further north. Thus a period such as the early Holocene, when there is higher boreal summer insolation and lower austral summer insolation than the present, has a boreal summer ITCZ location farther north and an austral summer ITCZ location farther south. This gives an expansion of the ITCZ.

**Figure 4:**
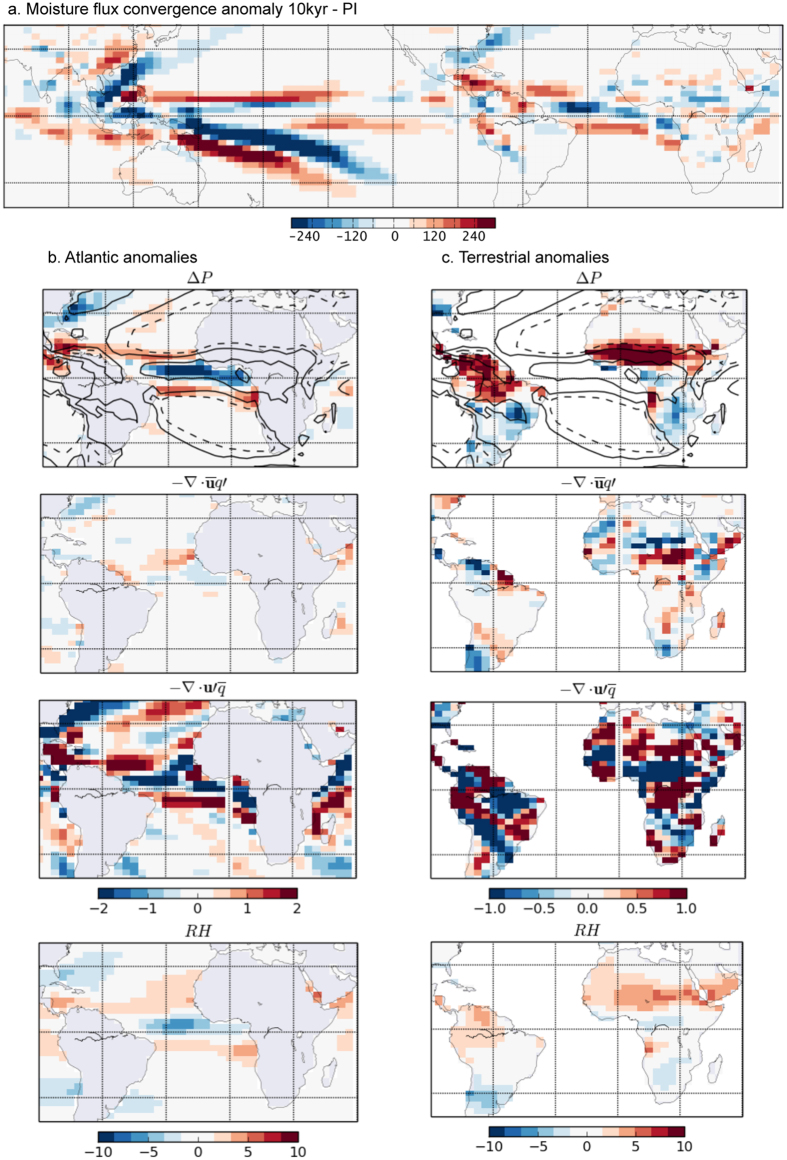
The relative importance of thermodynamic and dynamic drivers of changes in the rainbelt. (**a**) Anomalies in the column integrated moisture flux convergence (MFC; see Methods for details) field for 10 kyr minus PI (10^9^ kg s^−1^); (**b**) Atlantic Ocean basin anomalies for 10 kyr minus PI, and (**c**) terrestrial anomalies for 10kyr minus PI. The top plot shows the change in precipitation (mm day^−1^) with contours of PI annual mean rainfall (1, 2, 5, 10 mm day^−1^). The upper middle plot shows the thermodynamic component of the change in MFC, the bottom middle plot shows the dynamic component (mm day^−1^). The bottom plot shows the column averaged relative humidity anomalies (1000 to 200 mb). Figure maps created using Python 2.6.6 (www.python.org) on Linux.

The moisture flux convergence (MFC; see Methods) anomalies for 10 kyr minus PI (Fig. 4a) strongly resemble precipitation anomalies (Fig. 4b top) over the marine rainbelt, as found in previous studies^26^. The decomposition of the MFC anomalies^27^ into changes relating to specific humidity, with an unchanged circulation (i.e. the thermodynamic part; Fig. 4b middle), and the circulation, with an unchanged humidity distribution (i.e. the dynamic part; Fig. 4b bottom), demonstrates that over the Atlantic the dynamics are by far the dominant factor in the response of the rainbelt to insolation seasonality (the thermodynamic component becomes more important in response to glacial drivers, Fig. S7). These circulation-driven changes correlate well with the Atlantic interhemispheric temperature gradient (Figs S8 and S9) and describe the rainbelt variations better than the location of the maximum moist static energy, which has previously been proposed as initiating changes in the meridional circulation^28^. This may prove advantageous for further research into the past rainbelt variations, since temperatures can be inferred from palaeorecords, unlike energetic changes.

There are far fewer palaeorecords available with which to compare in marine regions than terrestrial regions. A recent marine proxy-based study^8^ inferred that the Atlantic ITCZ experienced meridional shifts, which contradicts the model response here. Having included these data in Fig. 2c (see also Fig. S3a), we suggest that the latitudes covered by the available palaeorecords^8^ may not yet extend far enough south to support or refute the modelled response of oceanic interhemispheric symmetry, highlighting the need for further data collection over marine areas. We did find that the majority of PMIP3 (Palaeoclimate Model Intercomparison Project 3) models display similar contraction/expansion in the Atlantic tropical rainbelt seasonal range as HadCM3 does, manifested as lower annual mean precipitation at the equator in the mid Holocene than the pre-industrial (Fig. S4 for PMIP models, and Fig. S14a for HadCM3).

### Continental variations

In contrast to the oceanic response to interhemispheric temperature gradients, the modelled continental rainbelt seems primarily influenced by local insolation. When summer insolation is lower, local land temperatures decrease more than SSTs, decreasing the land-sea temperature contrast (Fig. S6). The outcome is a reduced summer monsoon circulation and less moisture advected from ocean to land. Equally importantly, lower land temperatures lead to less convective activity and lower rainfall over much of the continent (and vice versa when summer insolation is higher; see Fig. 3b for Africa). The coherent continent-wide response of seasonal terrestrial precipitation to insolation is markedly different from the terrestrial convergence zone response, which undergoes sharp meridional shifts (Fig. S10). Unlike over the ocean, the large-scale fluxes of moisture as described by the MFC (Fig. 4a) do not explain the modelled terrestrial response of precipitation to insolation seasonality (Fig. 4c top). The precipitation anomalies resemble more closely the evaporation and column-averaged relative humidity anomalies (Fig. 4c bottom), indicating a dominance of local thermodynamic processes over terrestrial regions^12^.

Because local seasonal insolation dominates the magnitude of seasonal rainfall over land, the total annual mean precipitation varies in concert with the insolation during the season with the highest rainfall. Consequently terrestrial regions on average appear to display hemispherically asymmetric ‘shifts’ in the annual mean rainbelt (Fig. 1b). Other PMIP3 models demonstrate similar patterns to HadCM3, particularly over Africa (Supplementary Information section 2b). Such meridional shifts in the terrestrial rainbelt have long been discussed in the palaeodata literature^3, 5, 9^. At the extreme northern and southern limits of the terrestrial rainbelt the model variation over the glacial cycle agrees well with palaeodata (Fig. 2c; Fig. S3). However, while the overall picture from the model is one of oceanic latitudinal symmetry and terrestrial asymmetry, there is clear regional variety overprinting this general pattern.

### Regional diversity in response

Several regions display rainbelt variations that do not simply follow the general patterns described in the previous sections. Over the west coasts of Africa and South America the oceanic expansion/contraction pattern in the rainbelt is advected over land (Fig. 2c) in the model, leading to north-south symmetry in rainfall intensity^25^ that is at odds with the continental response further east. This symmetry was previously found in West African palaeodata^18^. As shown in Fig. 2c and Fig. S3, there is model-data agreement on the west coasts of the two continents in the timing of maximum Holocene rainfall occurring in the early Holocene both north and south of the equator; this is also seen in other PMIP3 models^25^ (Fig. S4). Over Africa there is some disagreement at the equator itself where the model suggests timing of maximum precipitation in the late Holocene whereas the data suggest the early Holocene (Fig. 2c: model region is blue whereas data point is orange). This may relate to the data integrating information from a catchment that is further north or that HadCM3 overestimates the advection of the ocean pattern or expresses the patterns at the wrong latitude, and is discussed in more detail in ref. 25.

**Figure 5:**
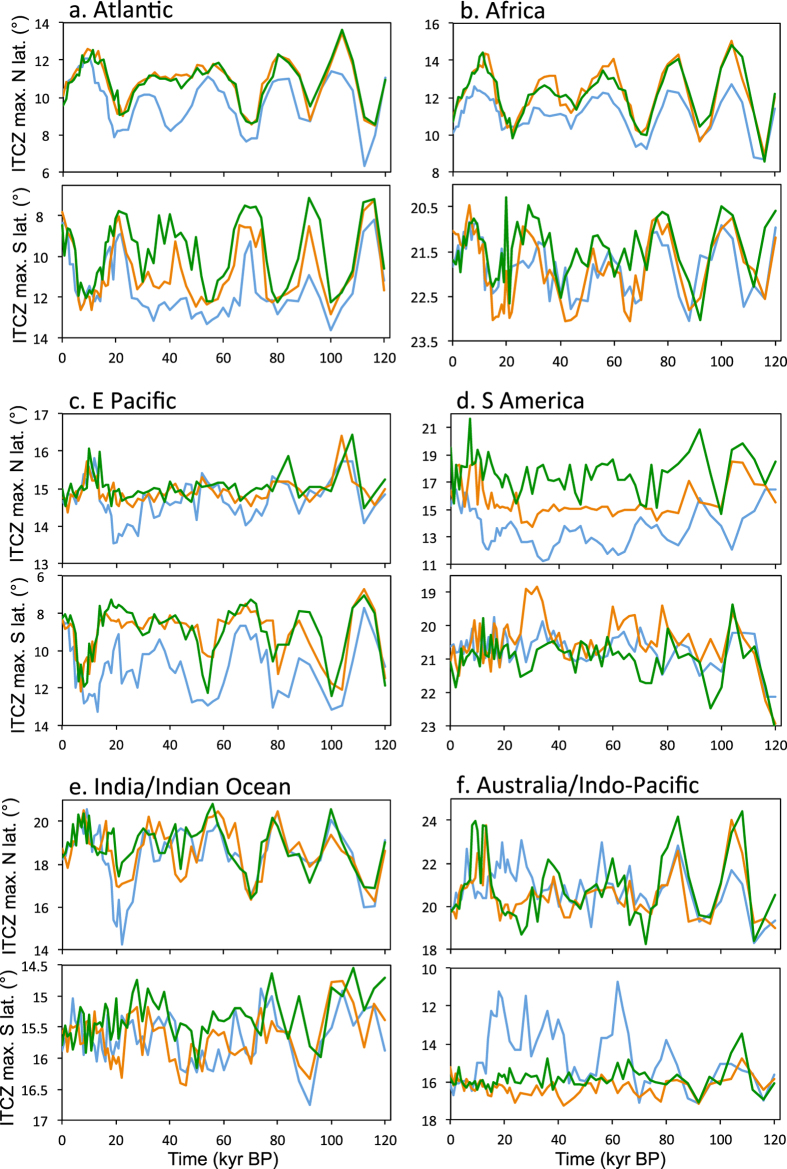
Regional variations in the tropical rainbelt. For each of six regions (Atlantic, Africa, East Pacific, South America, Indian Ocean, and Indo-Pacific) the northern hemisphere annual maximum northward extent (top) and southern hemisphere annual maximum southward extent (bottom) of the mean tropical rainbelt (ITCZ) for the ORB-ONLY (green), ORB+GHG (orange), and ALL (blue) set of simulations are displayed.

Over the Indo-Pacific/Australian regions the rainbelt is more highly sensitive to ice-sheets and sea level changes than orbital configuration (Fig. 5f). Here, there is significant local land exposure during the glacial, influencing pressure gradients that produce large-scale changes to the Walker circulation^29^. The E-W influence of the Walker circulation extends across the Indian Ocean to Eastern Africa. This contributes to muting the response to orbitally induced seasonality changes in the Indo-Pacific and the Indian Ocean, especially in the late glacial, when insolation variability is relatively small anyway. In addition, the interhemispheric temperature gradient in the Indian Ocean is weak and varies with 41 kyr cyclicity in the model, related to the obliquity cycle (as opposed to other ocean basins, which vary with a periodicity of ~21 kyr). This leads to the Indian Ocean maximum southerly extent of the rainbelt occurring at times of high obliquity (~42 kyr and 94 kyr; see Fig. S12).

Over northern South America, the maximum northern rainbelt latitude in the ALL experiment counter-intuitively varies in anti-phase with boreal summer insolation. As a result of local land being positioned to the south of the most northward extent of the rainbelt, it is drawn southward when summer insolation is high rather than northward (Figs 2a, 5d). Thus, over northern South America precipitation is higher at times when the local rainbelt is located further south (the opposite relationship to over northern Africa and India). This outcome occurs in the model only when ice-sheet forcing is included (the ALL experiment). The presence of the Laurentide ice-sheet changes the seasonal cycle of the rainbelt migration in that it forces more rapid movement south in the late summer/early autumn. Hence, precipitation earlier in the summer becomes more important for setting the maximum northerly seasonal excursion of the rainbelt than in the ORB-ONLY experiment, and at this point in the year there is a larger influence from land-sea temperature contrasts. Thus, ice-sheets modify the seasonality of precipitation, which alters the correlations between insolation forcing and rainbelt position (see Supplementary Information section 3 for a more detailed explanation).

One further notable feature in the marine rainbelt is that its equatorward movement is reduced every other precession cycle (at ~96 kyr and ~44 kyr BP; Fig. 5) when both obliquity is high (Fig. S7) and expanded ice-sheets are present. This is particularly noticeable in the S Atlantic (Fig. 5a). In this case, the presence of expanded ice-sheets shifts the convergence zone south and results in greater influence from high southern latitudes where the influence of obliquity is greater than precession. There is a resulting modification of the seasonal cycle of rainbelt migration that occurs only when obliquity is high (more detail in Supplementary Information section 4).

## Discussion

These regional responses demonstrate the potential pitfalls of equating local precipitation from palaeorecords with large-scale rainbelt shifts, and highlight the importance of global data syntheses for understanding the large-scale circulation. In order to effectively evaluate the modelled modes of multi-millennial variability described here, more palaeodata from tropical marine regions is essential to incorporate into such data synthesis.

We find that different modes and mechanisms of rainbelt movement are dominant over land and ocean, as well as at different points during the last glacial cycle. There are also significant local departures from the zonal mean response in our fully coupled ocean-atmosphere-vegetation model. In one region the intensity of precipitation even displayed an anti-correlation with the position of the local rainbelt. These findings complicate the classical paradigm of meridional Hadley circulation changes driven by cross-equatorial energy transport, as has been debated in recent studies^19, 30^. These are not just important considerations for mechanistic interpretations of palaeodata but also for future changes to the rainbelt.

## Methods

We performed 62 time slice simulations with the Hadley Centre coupled atmosphere-ocean-vegetation climate model, HadCM3^22–24^, coupled to the MOSES2.1 land surface scheme and the TRIFFID dynamic vegetation model. Four sets of simulations were conducted covering the last 120 000 years, at a frequency ranging from every 4000 years at the start (120–80 kyr BP), to 2000 years (80–22 kyr BP) and to every 1000 years (22–0kyr BP) (as in ref. 31 except for the inclusion of dynamic vegetation). Experiment **ALL** has glacial-interglacial changes to ice-sheet volume derived from sealevel reconstructions, and greenhouse gases, and orbital configuration changes described in ref. 32. Experiment **ORB+GHG** includes changes to orbital configuration and greenhouse gases, and **ORB−ONLY** has only changes to orbital configuration. In the fourth experiment, **HE**, 1Sv freshwater hosing was applied for 100 years to the N Atlantic (50–70°N) to simulate Heinrich events 1–6^31^ on top of the ALL boundary conditions. In the ALL, ORB+GHG, and ORB-ONLY simulations the initial conditions were based on a spun-up pre-industrial simulation and each was run for 500 years. The mean climatologies are of the last thirty years of each simulation.

We have used a climate model with coupled dynamic vegetation here. Previous sets of model experiments over the last glacial cycle have used a version with fixed vegetation^31^. The overall results presented in the paper, in terms of the temporal patterns of rainbelt variations, are not sensitive to whether the vegetation is fixed or dynamic.

To calculate the extreme range seasonally covered by the ITCZ we followed ref. 21. To estimate the northern limit of the core of the tropical precipitation belt at each longitude we used:

1 $$ITCZ(lon)=\frac{{\sum }_{y=lat(pr\,{\rm{\max }})}^{35^\circ N}pr(lon,y)lat(y)}{{\sum }_{y=lat(pr\,{\rm{\max }})}^{35^\circ N}pr(lon,y)}$$

where *lon* and *lat* stand for longitude and latitude respectively, *pr* stands for precipitation and *pr max* is the maximum precipitation. The same equation is used to calculate the southern limit of the ITCZ in winter, with a modified summation limit at 35°S latitude. The ITCZ latitudes were calculated at each model grid longitude for each time slice for each month, and then the maximum N/S latitudes (in any month) were used to define the ITCZ range for each time slice.

The column integrated moisture flux convergence (MFC) = $\int -\nabla .(q{\boldsymbol{u}})\mathrm{dp}$ where *q* is specific humidity and ***u*** is wind velocity. Both ***u*** and *q* can be linearised as, for example, ${\boldsymbol{u}}=\bar{{\boldsymbol{u}}}+{\boldsymbol{u}}^{\prime}$, where $\bar{{\boldsymbol{u}}}$ is the PI mean. Changes in the MFC from the PI can thus be decomposed into components, $-\nabla .\bar{{\boldsymbol{u}}}q^{\prime}$, that is due to the flux of anomalous humidity by the PI winds: the thermodynamic component. And $-\nabla .{\boldsymbol{u}}^{\prime} \bar{q}$, that is due to the flux of PI humidity distribution by the anomalous winds, the dynamic component. We ignore the cross term, $-\nabla .{\boldsymbol{u}}^{\prime} q^{\prime}$. See ref. 27 for full details of this derivation.

## Electronic supplementary material

Supplementary Information
