# Supplementary material for: Ocean dominated expansion and contraction of the late Quaternary tropical rainbelt
Source: Sci Rep. 2017 Aug 24;7:9382. doi: 10.1038/s41598-017-09816-8 (PMC5571209; doi:10.1038/s41598-017-09816-8)

Ocean dominated expansion and contraction of the late Quaternary tropical rainbelt: Supplementary Information

Joy S. Singarayer, Paul J. Valdes, William H. G. Roberts

1. Impact of Heinrich events vs. seasonal insolation

The model results suggest that the oceanic tropical rainbelt seasonal range responds to changes in insolation seasonality (primarily controlled by orbital precession) by expanding and contracting whereas the terrestrial response changes through meridional shifts in the rainbelt. At the northern limit in boreal summer, the land and ocean regions respond in concert, whereas the southern rainbelt limit in austral summer produces opposing ocean and land responses (see main text for details). In contrast, northern high latitude forcing via freshwater hosing (in order to approximate a Heinrich event) produces a homogeneous southward shift in the seasonal range over both land and ocean.

Concentrating on the southern hemisphere around the Atlantic and Africa, when austral summer insolation is lower local land temperatures decrease more than sea surface temperatures (SSTs), decreasing the land-sea temperature contrast (Fig. S6). The outcome is reduced monsoon circulation and lower moisture advected from ocean to land. In addition, there is less convective activity, lower relative humidity (Fig. S6), and lower rainfall over much of southern Africa. One can contrast this response to that of Heinrich event forcing (Fig. 2b). In austral summer, even though the land-sea temperature contrast is similarly reduced in southern Africa, it is because South Atlantic SSTs have increased (Fig. S6), following the bipolar seesaw mechanism. This results in greater moisture transport onto the continent, increasing humidity and rainfall over southern Africa, even though the monsoonal circulation is reduced. Consequently, the terrestrial rainbelt appears to shift southward in response to Heinrich event forcing.

The southern Atlantic responds to broader interhemispheric changes, which shift both the northern and southern limit of the Atlantic rainbelt southwards due to the latitudinally asymmetric extratropical forcing. The model rainbelt northern limit produces larger southerly shifts than the southern limit in response to the high northern latitude forcing, as can be seen in Fig. 2b.

The model patterns observed over the Atlantic and surrounding continents extend into the Indian Ocean and Easternmost Pacific the response is less clear in the Pacific as a whole. Likewise, other climate models demonstrate the strongest and clearest southerly shift of the tropical rainbelt over the Atlantic due to similar high northern latitude freshwater input, while there is a greater variation in response outside the Atlantic region[74].

2. Fidelity of HadCM3 results

2.1 Comparison of HadCM3 with palaeodata

The multi-millennial trends in precipitation from HadCM3 over the last glacial cycle have been compared with palaeodata (Table S1) time series that have been previously interpreted as representing changes to precipitation or hydroclimate in a broader sense. As shown in Fig. 2c of the main text we calculated the timing of maximum precipitation during the Holocene from the palaeodata records and for each grid cell of the model for comparison of the local phasing of precipitation variation. We found that at 81% of the points the model and data timing of maximum precipitation match to within 2kyr.

Fig. S3 demonstrates the full time series comparisons at eleven of the palaeodata locations. Where possible we chose records that cover more than one precessional cycle and include the majority of the Holocene time period. In most of the locations the models and data demonstrate not only similar phasing of precipitation, but also similar longer-term changes in the magnitude and rate of variations over the glacial cycle (e.g. Fig. S3h). There are locations where there is disagreement and one example of this is over East Africa (Fig. S3b). Here the models and data show

opposing trends. Model-data comparison over this region is discussed further in [25]. The differences partly relate to the low resolution of the model, which impacts the location of atmospheric boundaries over the high topography of the rift valley. In such cases it may be the case that climate patterns are produced that are shifted in their location from where they would occur in reality.

Similarly, over the Gulf of Guinea there is a mismatch in the timing of maximum precipitation during the Holocene (Fig. 2c), where in the data the maximum occurs in the early Holocene, whereas in the model it occurs in the late Holocene. Looking at the full available time series (Fig. S3k), it becomes clear that there is a better match in the phasing during the glacial period, and that the trends in model and data only really diverge during the Holocene[25]. This relates to the impact of the ice-sheet forcing in the model, which pushes the Atlantic ITCZ south during the glacial and leads to this particular location sitting in the northern part of the rainbelt that is in phase with northern hemisphere summer insolation, whereas in the Holocene (with less expansive ice-sheets) it sits in the central part of the rainbelt that is in anti-phase with northern summer insolation in the model (which doesn't match with the data). Overall, locations that are close to geographical boundaries of physical phenomena (relating to topographic or atmospheric circulation boundaries) are more likely to display poorer model-data comparisons. However, in general we demonstrate that there is a high level of model-data agreement in multi-millennial variations in hydroclimate over the last glacial cycle.

2.2 Comparison of HadCM3 with other climate models

There is a lack of simulations with other full complexity climate models that cover a whole glacial cycle. The Palaeoclimate Model Intercomparison Project 3 (PMIP3) provides the best opportunity for inter-model comparison of multi-millennial variation in the tropical rainbelt.

There are several key regions where the majority of climate model simulations contributed to the PMIP3 suite show similar changes in tropical precipitation. In the

mid-Holocene (MH; 6kyr BP) compared to the pre-industrial (PI; 0 kyr BP) all PMIP3 models demonstrate northward enhancement of precipitation over N Africa (Fig. S4) as does HadCM3 (Fig. S14a). This is also seen through calculations of the northern limit of the tropical rainbelt, where this is further north at the MH than PI for HadCM3 (Fig. 5) and the PMIP models[21,25]. Similarly, there is consistency in the movement of the southern limit of the rainbelt over Africa. Here, the majority of models (10 of 13 models) suggest the rainbelt southern limit to be further north in the MH than PI [Fig. 3b in [25]). The overall result is that there is a high level of model agreement that the African tropical rainbelt underwent a meridional shift south in its seasonal range between the MH and PI. The intermodel variation over central and Eastern parts of Africa is much higher.

The majority of PMIP3 models demonstrate expansion/contraction in the Atlantic tropical rainbelt seasonal range (Fig. S4) as HadCM3 does (Fig. S14a). This manifests as lower annual mean precipitation at the equator in the MH than PI and higher MH-PI precipitation to the north and south of this. The annual mean variation belies the variation in any one month/season, in which the Atlantic rainbelt shifts north/south (Fig. 3). Similarly, the boreal summer (JJAS) Atlantic tropical rainbelt in the last glacial maximum (LGM) shifts southwards in most PMIP3 models, but spatial patterns of anomalies are less consistent in boreal winter, leading to larger intermodel variation in the LGM-PI anomalies than MH-PI (Fig. S15 and Fig. S14b).

Regions where there is less consistency between models provide vital challenges to the palaeodata community to focus new data collection efforts and for overall synthesis, and for the palaeoclimate modelling community to understand the reasons for such divergences and for model optimization exercises. The degree of intermodel variation depends on the driving forces introduced. For example, the MH-PI precipitation anomalies over North Africa are highly consistent between models, whereas the LGM-PI response in the same region is one of the least consistent even in terms of the sign of the anomaly. Conversely, the Indo-Pacific

response displays low model agreement in the MH, but a greater agreement at the LGM. Similarly, the Eastern Pacific precipitation response at the MH is more variable than the southward shift that occurs at the LGM.

3. Seasonality in the northern limit of the South American rainbelt

The most northern position of the rainbelt over part of South America (280-300°E) during boreal summer displays interesting differences between the ORB-ONLY and ALL experiments. This is the region around the Cariaco Basin, where a run-off proxy record was interpreted as precipitation that was in phase with local summer (e.g. July) insolation, and thus that the ITCZ shifts were also in phase with local insolation. Our model results suggest that the northernmost ITCZ position of the ITCZ is, in general, in phase with local insolation and precipitation amount for ORB-ONLY, but in antiphase with local insolation and precipitation amount when ALL forcings are included (Fig. 5d top; Fig. S11). In the model the cause of this is a change in the timing of the most northward position of the rainbelt.

In the ORB-ONLY experiments the most northerly position of the rainbelt over S America occurs in September/October (Fig. S16, top). In this season the northerly position of the ITCZ varies in phase with local insolation. In the ALL experiment the most northerly extent occurs in July/August. During this season the ITCZ varies in anti-phase with local insolation, moving south when boreal insolation is high.

In the ALL experiment the October rainbelt is positioned much further south than in ORB-ONLY (Fig. S16, bottom). This is because the position of the rainbelt in October in the ALL experiment varies more in response to the ice sheet forcing than it does to insolation. When the Laurentide Ice Sheet expands the seasonal snow cover extends much further south and the resultant increase in the local latitudinal temperature gradient shifts the ITCZ south in Sept/Oct.

The reason for the anti-phasing of the location of the July/August rainbelt with insolation, which is seen in both experiments, is the configuration of the land: most

local land is to the south of the northernmost part of the July/August ITCZ. When local summer insolation is high, the land-sea temperature contrast increases and the atmospheric circulation intensifies, however, because the land is to the south of the ITCZ the rain belt moves south, apparently away from the warmer hemisphere, but in fact towards the locally warmed land. This mechanism occurs during July and August in the model in both the ORB-ONLY and ALL experiments. Therefore, when there is extensive Northern Hemisphere ice cover and the maximum northward extent to the ITCZ occurs in July/August, we see an anti-phase relationship with insolation. When there is no ice and the maximum northward extent occurs in September/October we see an in phase relationship. This has implications for the interpretation of proxies.

Regardless of whether there is ice present or not, the amount of rainfall in Northern S America shows an in phase relationship with insolation, increasing when the boraeal summer insolation increases (Fig S11). However, as we have just described, when ice is present the northward extent of the ITCZ can move south in response to boreal summer insolation. If, therefore, a proxy is sensitive to the maximum extent of the ITCZ, for example a record that lies at the very edge of the ITCZ's range, it cannot necessarily be interpreted in the context of changes in the total amount of rainfall in the ITCZ. Conversely, a proxy that is sensitive to the mean amount of rainfall, for example a record in the core of the ITCZ, should not be interpreted in the context of shifts in the ITCZ

Whether or not the modelled response and the actual climate's response to insolation are the same in the exact detail, this result demonstrates the potential for over- or mis- interpreting hydroclimate shifts from single proxy records.

4. Southern Atlantic rainbelt and obliquity

A notable feature over the marine southern rainbelt limit (Fig. 1c; bottom) is that while the ORB-ONLY experiment displays precessionally influenced 21-kyr cyclicity,

the ALL experiment equatorward movement of the rainbelt is reduced every other precession cycle (at ~96 kyr and ~44 kyr BP), when obliquity is also high (Fig. S12). This response is most prominent in the southern Atlantic (Fig. 3).

During these times, when both obliquity is high and expanded ice-sheets are present, there is a change in the seasonality of the rainbelt movement such that the maximum southern latitude occurs in April rather than in February. (Fig. S9d and Fig S13). The latitudinal pattern of austral summer mean rainfall at these times (Fig. S9a; 44 kyr) is more similar to that of time slices with low summer insolation (Fig. S9a, 104kyr) than high insolation (Fig. S9a, 22kyr, 70kyr). The influence of ice-sheets on the interhemispheric temperature gradients forces the zone of convergence and uplift to move south (Fig. 1c) resulting in greater influence from high southern latitudes. When obliquity is high there is larger south Atlantic warming at high southern latitudes vs. mid southern latitudes in austral summer (Fig. S9). Sensible and latent heat flux increase more at extratropical southern latitudes and shifts the zone of convergence further south. Cloud cover and rainfall occur further south and earlier in the austral summer (Fig. S9).

There are low correlations with global interhemispheric radiative balance or global temperature gradients, particularly when ice-sheet forcing is introduced. However, the movement of the rainbelt correlates most highly with the regional tropical interhemispheric temperature gradient (Fig. S9b and e), influenced by both land and ocean, but with increased oceanic influence when ice-sheets are included (Fig. S9e) because of the overall southward shift in the ITCZ, where there is a lower proportion of land. The findings suggest that while the terrestrial rainbelt responds to local changes in insolation and other forcings the oceanic rainbelt responds to regional interhemispheric gradients.

References

33. Wang, Y.J., H. Cheng, R.L. Edwards, Z.S. An, J.Y. Wu, C.-C. Shen, and J.A. Dorale. A High-Resolution Absolute-Dated Late Pleistocene Monsoon Record from Hulu Cave, China. *Science*, 294, 2345-2348 (2001).
34. Wang, Y., H. Cheng, R.L. Edwards, Y. He, X. Kong, Z. An, J. Wu, M.J. Kelly, C.A. Dykoski, and X. Li. The Holocene Asian Monsoon: Links to Solar Changes and North Atlantic Climate. *Science*, 308, 854-857 (2005).
35. Dong, J., Y. Wang, H. Cheng, B. Hardt, R.L. Edwards, X. Kong, J. Wu, S. Chen, D. Liu, X. Jiang, and K. Zhao. A high-resolution stalagmite record of the Holocene East Asian monsoon from Mt Shennongjia, central China. *The Holocene*, 20, 257-264 (2010).
36. Wang, X., A.S. Auler, R.L. Edwards, H. Cheng, E. Ito, Y. Wang, X. Kong, and M. Solheid. Millennial-scale precipitation changes in southern Brazil over the past 90,000 years. *Geophys. Res. Lett*., 34, L23701 (2007).
37. van Breukelen, M.R., H.B. Vonhof, J.C. Hellstrom, W.C.G. Wester, and D. Kroon. Fossil dripwater in stalagmites reveals Holocene temperature and rainfall variation in Amazonia. *Earth and Planetary Science Letters*, 275, 54-60 (2008).
38. Griffiths, M.L., R.N. Drysdale, M.K. Gagan, J.-x. Zhao, L.K. Ayliffe, J.C. Hellstrom, W.S. Hantoro, S. Frisia, Y.-x. Feng, I. Cartwright, E. St. Pierre, M.J. Fischer, and B.W. Suwargadi. Increasing Australian-Indonesian monsoon rainfall linked to early Holocene sea-level rise. *Nature GeoScience*, 2, 597-664 (2009).
39. Weldeab, S., Lea, D.W., Schneider, R.R., Andersen, N. 155,000 years of West African monsoon and ocean thermal evolution. *Science*, 316, 1303-1307 (2007).
40. Schmidt, M. W., Spero, H. J. & Lea, D. W. Links between salinity variation in the Caribbean and North Atlantic thermohaline circulation. *Nature* 428, 160_163 (2004).

41. Lyons R., Tooth S., Duller G.A.T. Late Quaternary climatic changes revealed by luminescence dating, mineral magnetism and diffuse reflectance spectroscopy of river terrace palaeosols: a new form of geoproxy data for the southern African interior. *Quaternary Science Reviews*, 95, 43-59 (2014).
42. Clemens, S. C., and Prell, W. L. A 350,000 year summer-monsoon multi-proxy stack from the Owen Ridge, Northern Arabian Sea. *Marine Geology*, 201, 35-51 (2003).
43. Beaufort, L., Kaars, S., Bassinot, F. C., and Moron, V. Past dynamics of the Australian monsoon: precession, phase and links to the global monsoon concept. *Climate of the Past*, 6, 695-706 (2010).
44. Schefuß, E., Schouten, S., Schneider, R.R. Climatic controls on central African hydrology during the past 20,000 years. *Nature*, 437, 1003-1006 (2005).
45. Tierney, J. E., et al. Northern hemisphere controls on tropical southeast African climate during the past 60,000 years. *Science* **322**, 252-255 (2008).
46. Berke, M.A., Johnson, T.C., Werne, J.P., Grice, K., Schouten, S., Sinninghe Damstê, J.S. Molecular records of climate variability and vegetation response since the Late Pleistocene in the Lake Victoria basin, East Africa. Quat. Sci. Rev. 55, 59-74 (2012).
47. Schefuß, E., Kuhlmann, H., Mollenhauer, G., Prange, M., Pätzold, J., Forcing of wet phases in southeast Africa over the past 17,000 years. Nature 480, 509-512 (2011).
48. Verschuren, D., Sinninghe Damst_e, J.S., Moernaut, J., et al. Half-precessional dynamics of monsoon rainfall near the East African Equator. Nature 462, 637-641 (2009).
49. Shiau, L. J., Chen, M. T., Huh, C. A., Yamamoto, M., & Yokoyama, Y. Insolation and cross-hemispheric controls on Australian monsoon variability over the past 180 ka: new evidence from offshore southeastern Papua New Guinea. *Journal of Quaternary Science*, 27, 911-920 (2012).

50. Bar-Matthews, M., A. Ayalon, M. Gilmour, A. Matthews, and C.J. Hawkesworth Sea-land oxygen isotopic relationships from planktonic foraminifera and speleothems in the Eastern Mediterranean region and their implication for paleorainfall during interglacial intervals, Geochimica et Cosmochimica Acta, 67, 17, 3181-3199 (2003).
51. Dutt, S., A. K. Gupta, S. C. Clemens, H. Cheng, R. K. Singh, G. Kathayat, and R. L. Edwards Abrupt changes in Indian summer monsoon strength during 33,800 to 5500 years B.P., Geophys. Res. Lett., 42, 5526–5532 (2015)
52. Holmgren, K., J.A. Lee-Thorp, G.R.J. Cooper, K. Lundblad, T.C. Partridge, L. Scott, R. Sithaldeen, A. Siep Talmaf and P.D. Tyson. Persistent millennial-scale climatic variability over the past 25,000 years in Southern Africa. Quaternary Science Reviews, 22, 2311-2326 (2003).
53. Asmerom, Y., V. Polyak, S. Burns, and J. Rassmussen Solar forcing of Holocene climate: New insights from a speleothem record, southwestern United States. Geology, 35, 1-4 (2007).
54. Partin, J.W., K.M. Cobb, J.F. Adkins, B. Clark, and D.P. Fernandez. Millennial-scale trends in west Pacific warm pool hydrology since the Last Glacial Maximum. Nature, 449, 452-455 (2007).
55. Frumkin, A., D.C. Ford, and H.P. Schwarcz. Continental Oxygen Isotopic Record of the Last 170,000 Years in Jerusalem. Quaternary Research, 51, 317-327 (1999).
56. Tjallingii, R., Claussen, M., Stuut, J.B.W., Fohlmeister, J., Jahn, A., Bickert, T., Lamy, F., Röhl, U. Coherent high- and low-latitude control of the northwest African hydrological balance. Nat. Geosci. 1, 670-675 (2008).
57. Partridge, T.C., deMenocal, P.B., Lorentz, S.A., Paiker, M.J., Vogel, J.C. Orbital forcing of climate over South Africa: a 200,000-year rainfall record from the Pretoria Saltpan. Quaternary Science Reviews, 16, 1125-1133 (1997).

58. Tierney, J.E., DeMenocal, P.B. Abrupt shifts in Horn of Africa hydroclimate since the last glacial maximum. Science, 342, 843-846 (2013).
59. Costa, K., Russell, J., Konecky, B., Lamb, H. Isotopic reconstruction of the African Humid Period and Congo Air Boundary migration at Lake Tana, Ethiopia. Quat. Sci. Rev. 83, 58-67 (2014).
60. Konecky, B.L., Russell, J.M., Johnson, T.C., Brown, E.T., Berke, M.A., Werne, J.P., Huang, Y. Atmospheric circulation patterns during late Pleistocene climate changes at Lake Malawi, Africa. Earth & Planetary Science Letters 312, 318–326 (2011).
61. Peck, J.A., Green, R.R., Shanahan, T., King, J.W., Overpeck, J.T., Scholz, C.A. A magnetic mineral record of Late Quaternary tropical climate variability from Lake Bosumtwi, Ghana. Palaeogeogr. Palaeoclimatol. Palaeoecol. 215, 37-57 (2004).
62. Chase, B.M., Meadows, M.E., Scott, L., Thomas, D.S.G., Marais, E., Sealy, J., Reimer, P.J. A record of rapid Holocene climate change preserved in hyrax middens from southwestern Africa. Geology 37, 703-706 (2009).
63. Collins, J.A., Schefuß, E., Govin, A., Mulitza, S., Tiedemann, R. Insolation and glacial-interglacial control on southwestern African hydroclimate over the past 140000 years. Earth Planet. Sci. Lett., 398, 1-10 (2014).
64. Armitage, S. J., Bristow, C. S., & Drake, N. A. West African monsoon dynamics inferred from abrupt fluctuations of Lake Mega-Chad. *Proceedings of the National Academy of Sciences*, 112, 8543-8548 (2015).
65. Stríkis, N. M., Cruz, F. W., Cheng, H., Karmann, I., Edwards, R. L., Vuille, M., Wang X., de Paula M.S., Novello V.F., and Auler, A. S. Abrupt variations in South American monsoon rainfall during the Holocene based on a speleothem record from central-eastern Brazil. *Geology*, 39, 1075-1078 (2011).
66. Cruz, F. W., Vuille, M., Burns, S. J., Wang, X., Cheng, H., Werner, M., Edwards R.L., Karmann I., Auler A.S., Nguyen, H. Orbitally driven east–west

antiphasing of South American precipitation. *Nature Geoscience*, 2, 210-214 (2009).

67. Covich, A., & Stuiver, M. Changes in oxygen 18 as a measure of long-term fluctuations in tropical lake levels and molluscan populations1. *Limnology and Oceanography*, 19, 682-691 (1974).
68. Hodell, D. A., Curtis, J. H., Jones, G. A., Higuera-Gundy, A., Brenner, M., Binford, M. W., & Dorsey, K. T. Reconstruction of Caribbean climate change over the past 10, 500 years. *Nature*, 352, 790-793 (1991).
69. Denniston, R. F., Wyrwoll K-H, Polyak V. J., Brown J. R., Asmerom Y., Wanamaker A.D., LaPointe Z. Ellerbroek, R. Barthelmes M., Cleary D., John Cugley, Woods D. Humphreys W.F. A stalagmite record of Holocene Indonesian–Australian summer monsoon variability from the Australian tropics. *Quaternary Science Reviews,* 78, 155-168 (2013).
70. Cheng, H., Sinha A., Cruz F.W., Wang X., Edwards R.L., d'Horta F.M., Ribas C.C., Vuille M., Stott L.D., and Auler A.S.. Climate change patterns in Amazonia and biodiversity. *Nature communications,* 4, 1411 (2013).
71. Thompson, L. G., Davis, M. E., Mosley-Thompson, E., Sowers, T. A., Henderson, K. A., Zagorodnov, V. S., ... & Francou, B. A 25,000-year tropical climate history from Bolivian ice cores. *Science*, 282, 1858-1864 (1998).
72. Baker, P. A., Rigsby, C. A., Seltzer, G. O., Fritz, S. C., Lowenstein, T. K., Bacher, N. P., and Veliz, C. Tropical climate changes at millennial and orbital timescales on the Bolivian Altiplano. *Nature*, 409, 698-701 (2001).
73. Bertaux, J., Sondag F., Santos R., Soubiès F., Causse C., Plagnes V., Le Cornec F., and Seidel A. Paleoclimatic record of speleothems in a tropical region: study of laminated sequences from a Holocene stalagmite in Central–West Brazil. *Quaternary International* 89, 1, 3-16 (2002).
74. Kageyama, M., Merkel, U., Otto-Bliesner, B., Prange, M., Abe-Ouchi, A., Lohmann, G., Ohgaito, R., Roche, D. M., Singarayer, J., Swingedouw, D., and

X Zhang: Climatic impacts of fresh water hosing under Last Glacial Maximum conditions: a multi-model study. *Clim. Past*, 9, 935-953 (2013).

Table S1. Details of the palaeorecords used in the synthesis map of Fig. 1c in the main text.

Record Name	Lon. (°**E)**	Lat. (°**N)**	Data source	Reference
Hulu Cave	119.16	32.5	$\delta^{18}O$ speleothem	Wang et al., 2001 (33)
Dongge Cave	108.08	25.28	$\delta^{18}O$ speleothem	Wang et al., 2005 (34)
Sanbao Cave	110.433	31.667	$\delta^{18}O$ speleothem	Dong et a., 2010 (35)
Botuvera	-49.16	-27.22	$\delta^{18}O$ speleothem	Wang et al., 2007 (36)
Cueva del Tigre Perdido	-77.31	-594	$\delta^{18}O$ speleothem	van Breukelen et al., 2008 (37)
Qunf	54.3	17.17	$\delta^{18}O$ speleothem	Fleitmann et al., 2007 (10)
Liang Luar	120.43	-8.53	$\delta^{18}O$ speleothem	Griffiths et a., 2009 (38)
Cariaco Basin	-65.2	10.7	ODP site 1002 %Ti	Haug et al., 2001 (4)
VM25-59	-33.5	1.4	Mg/Ca and d18O planktic forams (SST/SSS)	Arbuszewski et al 2013 (8)
VM30-40	-23	0	Mg/Ca and d18O planktic forams (SST/SSS)	Arbuszewski et al 2013 (8)
RC24-11	-11.3	-2	Mg/Ca and d18O planktic forams (SST/SSS)	Arbuszewski et al 2013 (8)
MD03-2707, Gulf of Guinea	9.4	2.5	Ba/Ca (riverine runoff)	Weldeab et al., 2007 (39)
Caribbean Sea	-76	12	$\delta^{18}O$ seawater	Schmidt et al., 2004 (40)
Erfkroon, Modder River	25.5	-28.8	Mineral magnetic and diffuse reflectance spectroscopy (DRS)	Lyons et al., 2014 (41)
Northern Arabian Sea	61	22	Ocean sediment stacked monsoon factor	Clemens and Prell, 2003 (42)
MD98-2175, Eastern Banda Sea	133.4	-5	Multi-proxy coccolith and pollen data	Beaufort et al., 2010 (43)
Congo River	11.47	-5.94	C_{29} δDwax	Schefuβ et al., 2005 (44)

Mouth				
Lake Tanganyika	29.83	-6.71	C_{28} δDwax	Tierney et al., 2008 (45)
Lake Victoria	33.20	-1.23	C_{28} δDwax	Berke et al., 2012 (46)
GeoB9307-3, Zambezi Delta	37.38	-18.56	C_{28} δDwax	Schefuβ et al., 2011 (47)
Lake Challa	37.7	-3.3	BIT index	Verschuren et al., 2009 (48)
Offshore Papua New Guinea	149	-11	^{232}Th activity flux/ $\delta^{18}O$	Shiau et al., 2012 (49)
Soreq Cave	35.03	31.45	$\delta^{18}O$ speleothem	Bar-Matthews et al., 2003 (50)
Mawmulh Cave, India	92	25	$\delta^{18}O$ speleothem	Dutt et al., 2015 (51)
Cold Air Cave	29.11	-24.02	$\delta^{18}O$ speleothem	Holmgren et al., 2003 (52)
Pink Panther Cave	-105.2	32.1	$\delta^{18}O$ speleothem	Asmerom et al., 2007 (53)
Gunung Cave	114.8	4.03	$\delta^{18}O$ speleothem	Partin et al., 2007 (54)
Jerusalem West Cave	35.15	31.78	$\delta^{18}O$ speleothem	Frumkin et al., 1999 (55)
GeoB7920-2, Offshore Mauritania	-18.58	20.75	Continental humidity index	Tjallginii et al., 2008 (56)
Tswaing Crater	28.75	-25.56	Grain size	Partridge et al., 1997 (57)
Gulf of Aden	44.3	12	C_{30} δDwax	Tierney and DeMenocal, 2013 (58)
Lake Tana	37.35	12	C_{28} δDwax	Costa et al., 2014 (59)
Lake Malawi	34	-10	C_{28} δDwax	Konecky et al., 2011 (60)
Lake Bosumtwi	-1.41	6.51	% LOI organic content	Peck et al., 2004 (61)
Spitzkoppe	15.18	-21.82	Hyrax midden $\delta^{15}N$	Chase et al., 2009 (62)
MD08-3167, Offshore Namibia	12.38	-23.32	C_{31} δDwax	Collins et al., 2014 (63)
Lake Chad	12-18	11-17	Lake shoreline dates	Armitage et al., 2015 (64)
Lapa Grande Cave	-44.3	-14.4	$\delta^{18}O$ speleothem	Strikis et al., 2011 (65)
Rio Grande do Norte	-37.7	-5.5	$\delta^{18}O$ speleothem	Cruz et al., 2009 (66)

Laguna Chichancanab	-88.75	19.8	$\delta^{18}O$ lake and Mollusca	Covich and Stuiver, 1974 (67)
Lake Miragoane	-73	17	$\delta^{18}O$ ostracoda	Hoddell et al., 1991 (68)
Cave KNI-51, Eastern Kimberley	128	-15	$\delta^{18}O$ speleothem	Denniston et al., 2013 (69)
Paraiso Cave	-55.45	-4.07	$\delta^{18}O$ speleothem	Cheng et al., 2013 (70)
Sajama ice core	-69	-18	$\delta^{18}O$ ice core	Thompson et al., 1998 (71)
Lake Uyuni	-68	-20	Gamma	Baker et al., 2001 (72)
Diamente	-77.5	-5.73	$\delta^{18}O$ speleothem	Cheng et al., 2013 (70)
Joao Arruda Cave	-57	-21	$\delta^{18}O$ speleothem	Bertaux et al., 2002 (73)

Fig. S1 The major modes of variability in precipitation by Empirical Orthogonal Function (EOF) analysis. (a) The first principal component (top) and corresponding EOF spatial pattern (bottom) for the global tropical marine rainbelt annual precipitation in the ORB-ONLY experiment. This describes 63% of the total variance in the data. (b) Same as (a) but the first principal component and EOF pattern for the global terrestrial rainbelt precipitation, accounting for 58% of the variance. [Figure maps created using Python 2.6.6 (www.python.org) on Linux]

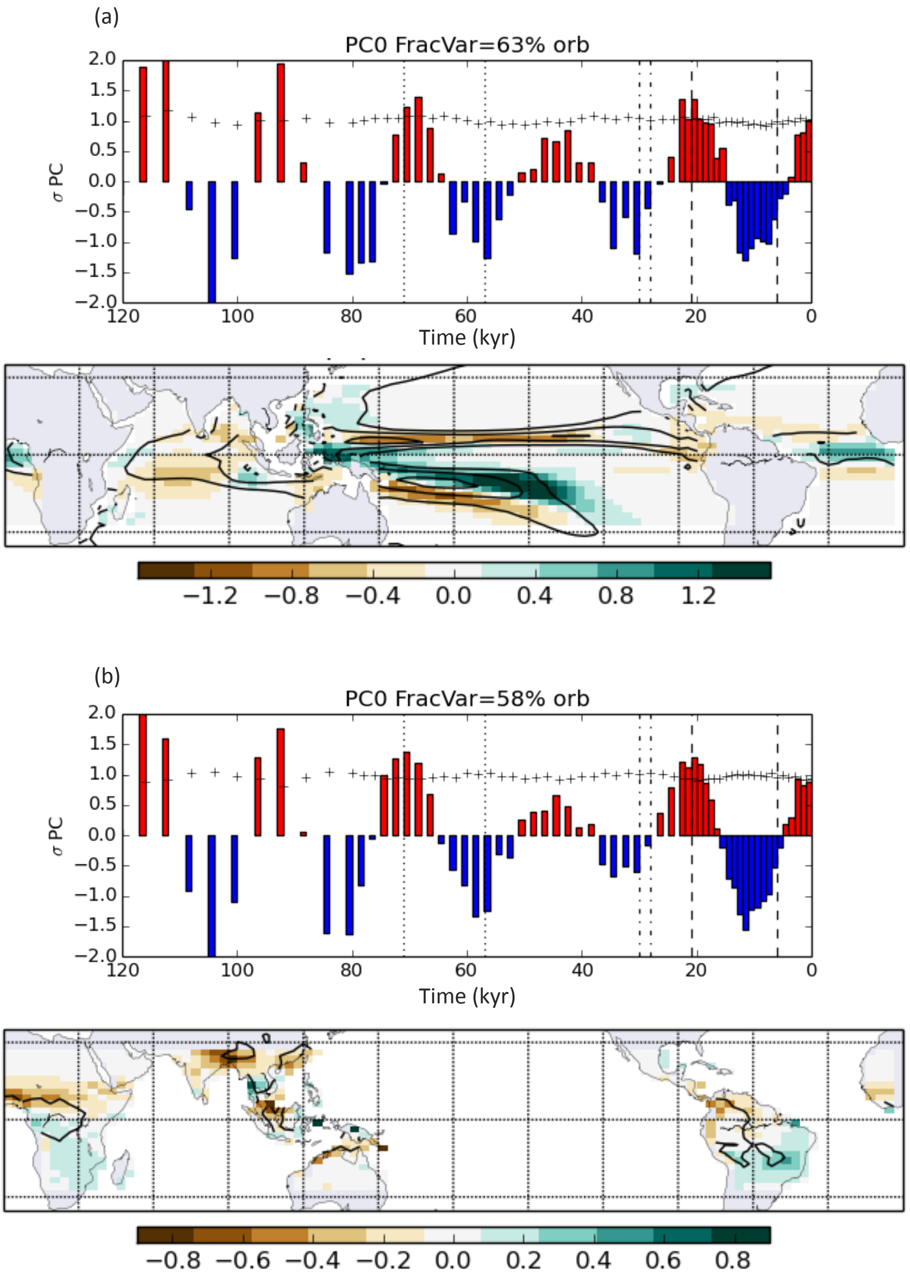

Fig. S2 Empirical Orthogonal Function (EOF) analysis of the Atlantic Ocean and surrounding land modelled precipitation. (a) The first principal component (left) and EOF spatial pattern for ORB-ONLY annual mean precipitation over the Atlantic. (b) Same as (a) but for ORB-ONLY terrestrial precipitation. (c) Same as (a) but for the ALL experiment. (d) Same as (b) but for the ALL experiment.
[Figure maps created using Python 2.6.6 (www.python.org) on Linux]

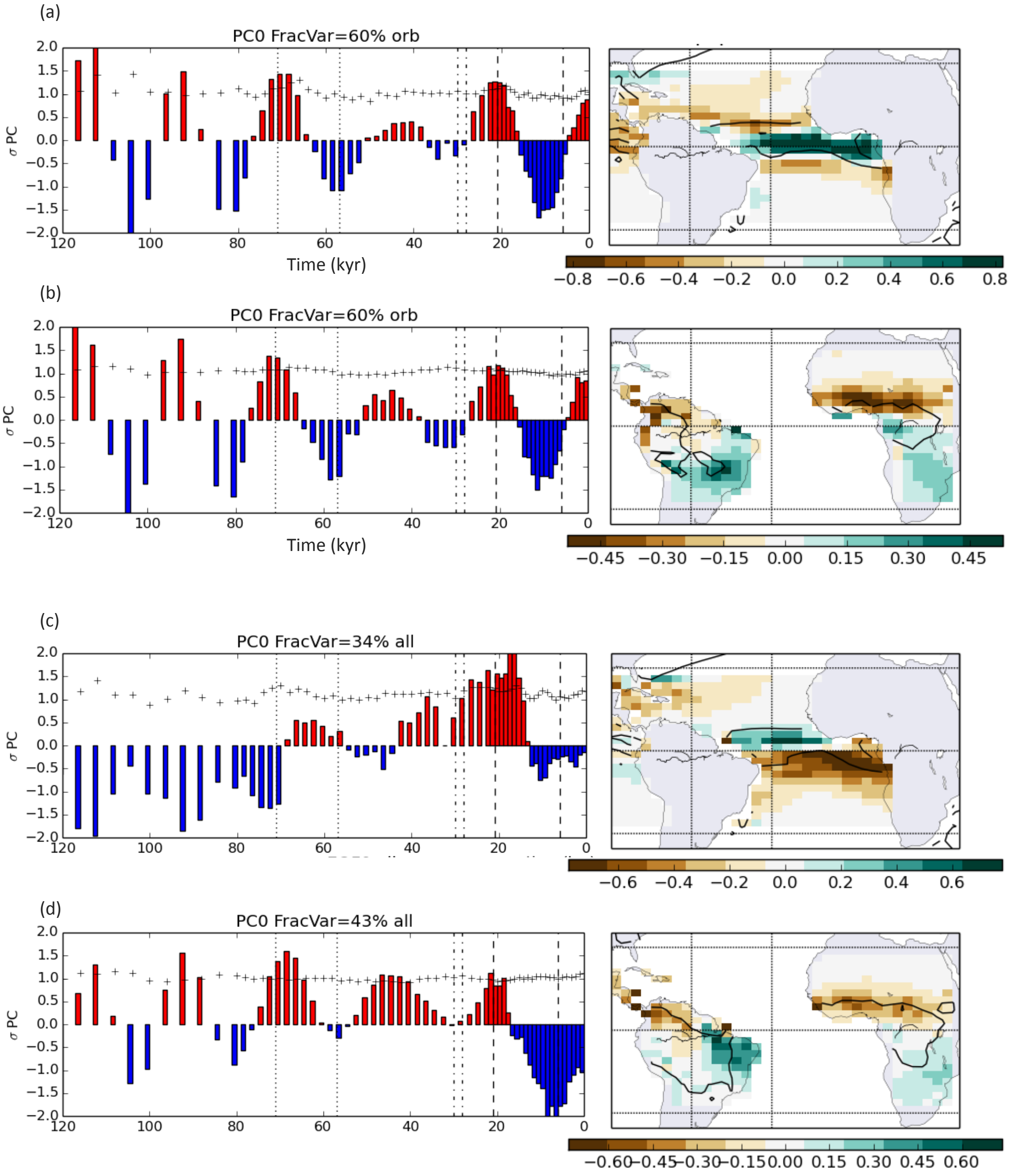

Fig. S3 Comparisons of modelled annual mean precipitation from the ALL experiment with palaeorecord proxies of hydroclimate. All time series plots (a, b, c, d, e, g, h, I, j, k, l) have right hand y-axes related to the model precipitation (sold black lines) at the location of each palaeodata record indicated by the arrows to the central map, with units of mm day^{-1} and x-axes units of kyr before present. The left hand y-axes are related to the palaeodata (dashed grey lines) in the units specified on each sub-plot label. The reference number in square brackets gives the source of each palaeodata record. The central map plot (f) is of annual mean precipitation anomalies for mid-Holocene (6 kyr BP) minus pre-industrial (0 kyr BP).
[Figure map created using Panoply (http://www.giss.nasa.gov/tools/panoply/) version 4.3.1]

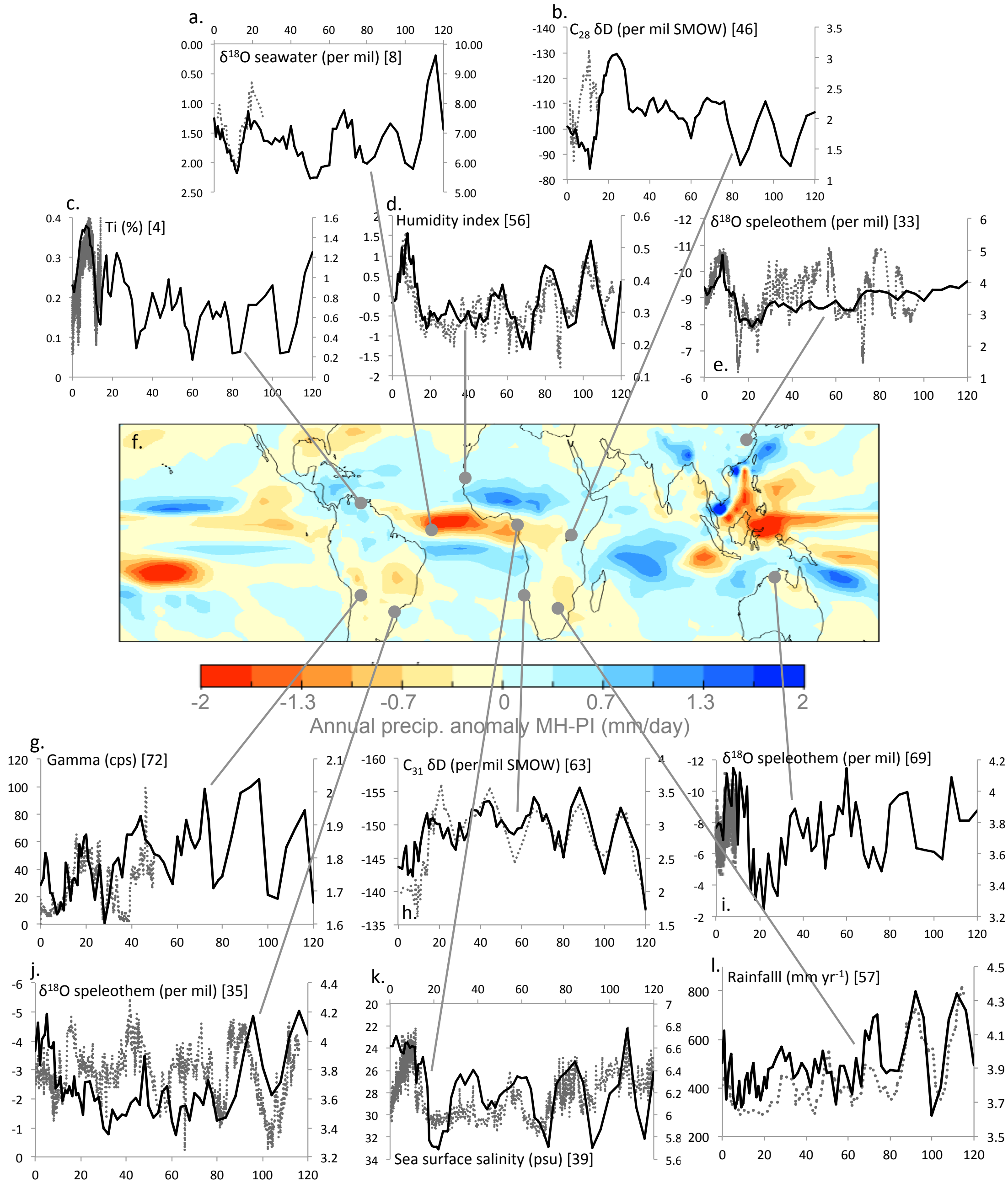

Fig. S4 Mid-Holocene (6 kyr BP) minus pre-industrial simulated annual mean precipitation anomalies from a variety of global climate models used in the Paleoclimate Model Intercomparison Project 3 (PMIP3).
[Figure maps created using Panoply (http://www.giss.nasa.gov/tools/panoply/) version 4.3.1]

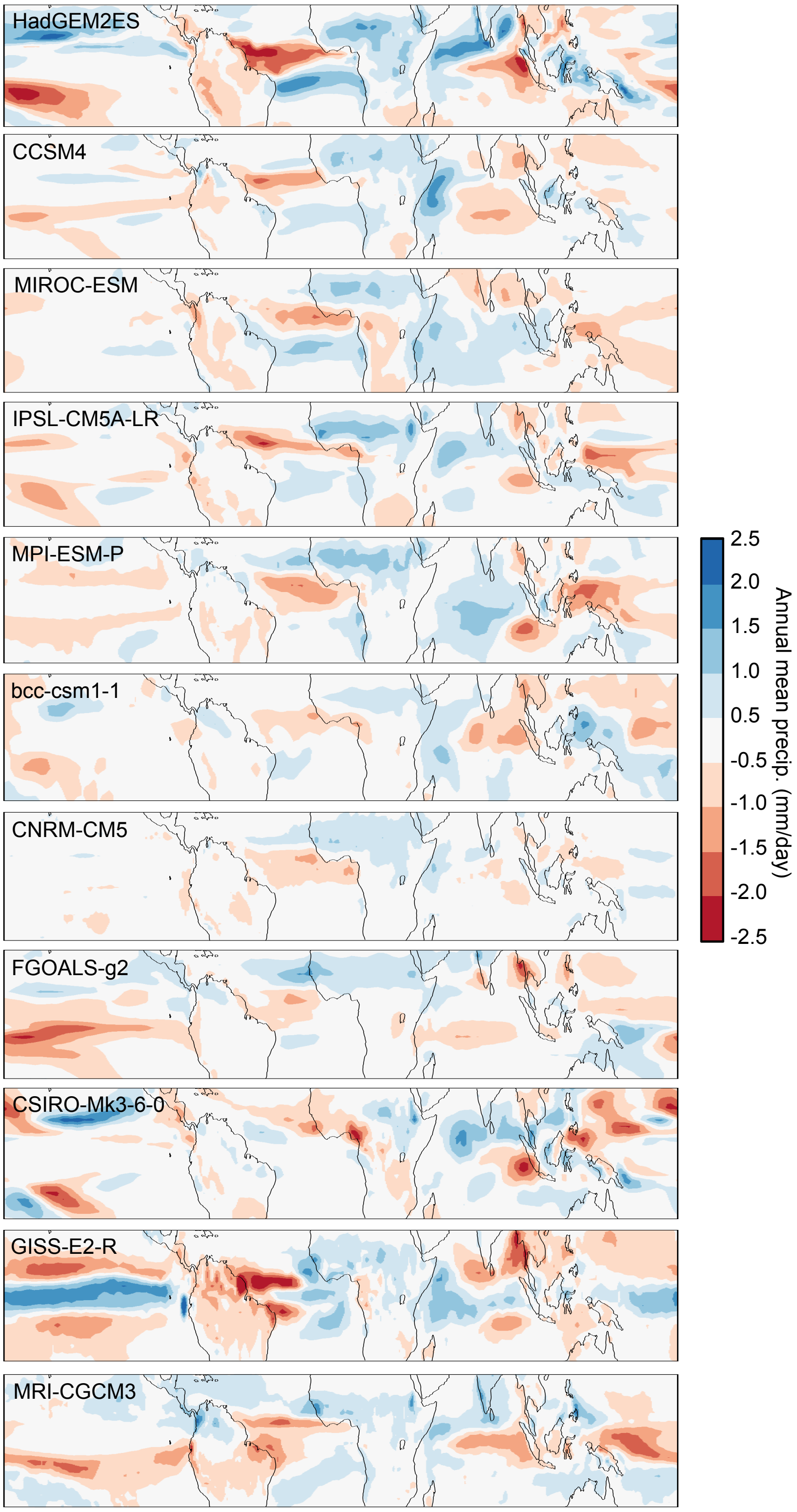

Fig. S5 Comparison of Atlantic precipitation (grey solid line) and Sea Surface Salinity (SSS; black dotted line) at various equatorial latitudes from 10-12°N (top) to 3-5°S (bottom) for the ORB-ONLY experiment (left) and ALL experiment (right) at 330-345°E. Arbuszewski et al (2013)[8] use primarily SSS palaeoproxy data to derive interpretations of Atlantic precipitation intensity (inversely related to SSS) and hence ITCZ position. The model demonstrates the same general inverse relationship between SSS and precipitation. It is especially evident in the ORB-ONLY experiment, but is significantly reduced in the ALL experiment.

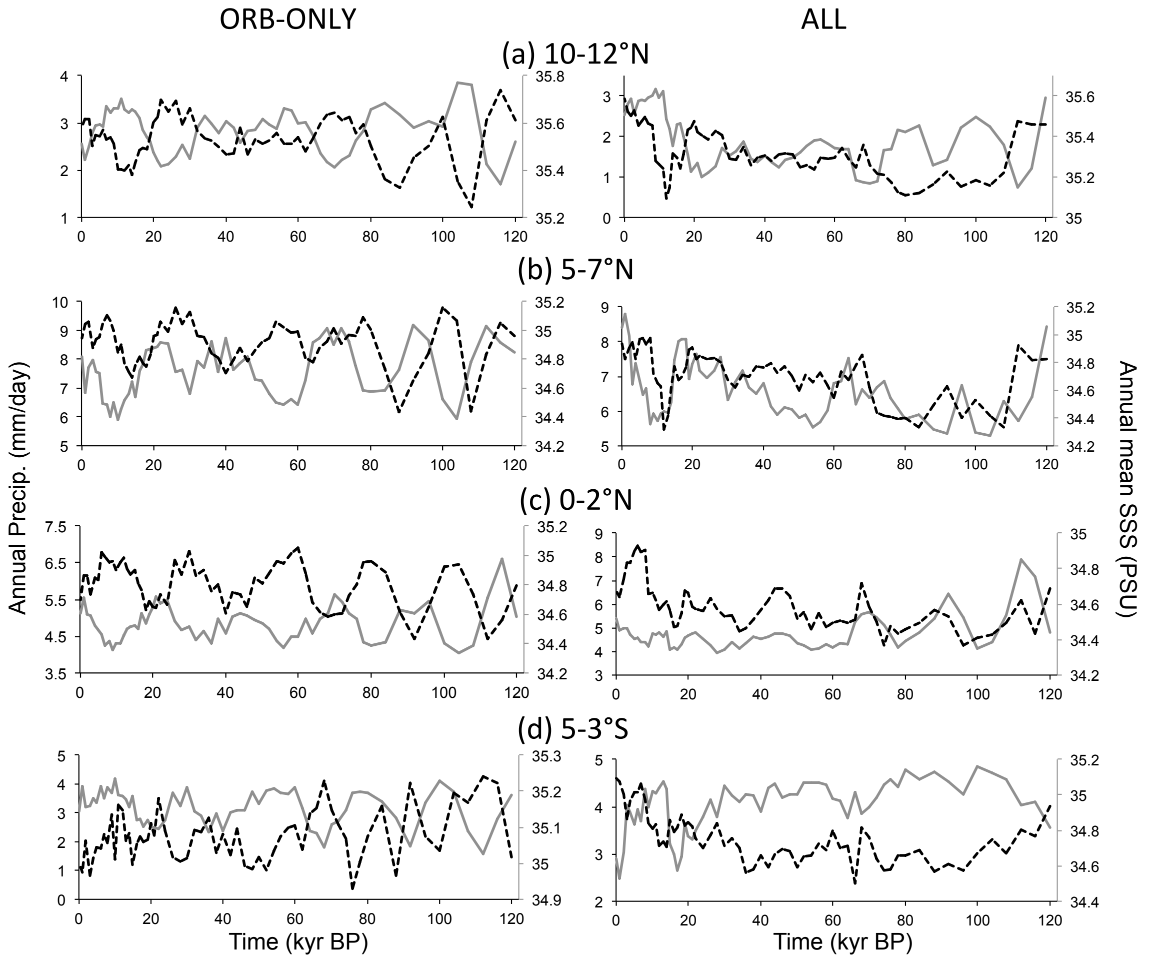

Fig. S6 Austral summer (DJF) climate anomalies (A) 10 kyr minus PI for the ALL experiment, and (B) Heinrich event 1 (HE1) minus related 17 kyr control simulation. [Created using NCL (https://www.ncl.ucar.edu) version 6.2.1]

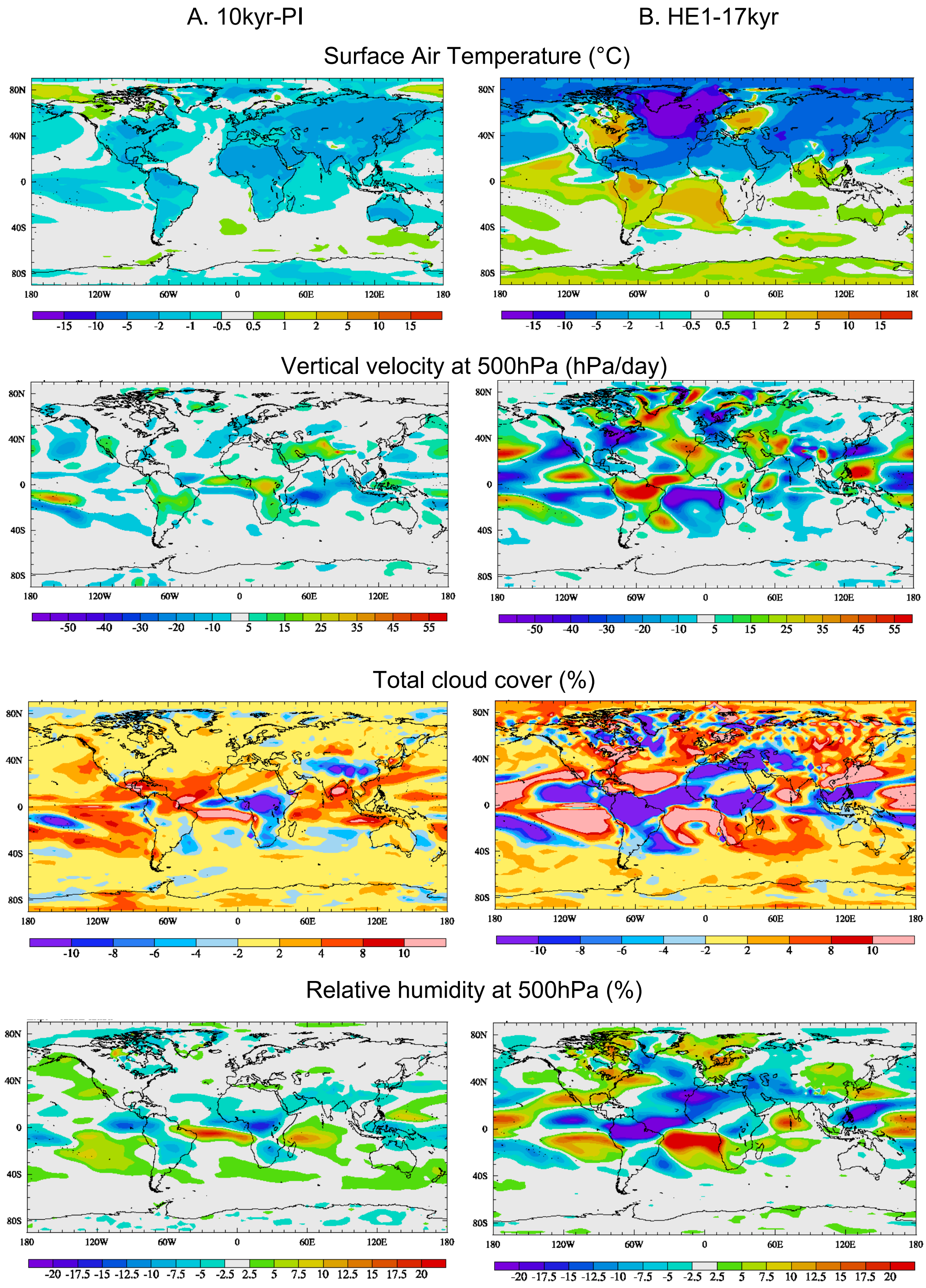

Fig. S7 Moisture flux convergence anomalies and decomposition for the 21kyr time slice in the ALL experiment. (a) Moisture flux convergence anomaly for the Atlantic Ocean region. (b) Advection component of the moisture flux convergence anomalies. (c) Convergence component of the moisture flux convergence. At the glacial maximum (21kyr) there is an increased importance of thermodynamics (plot b) in addition to dynamics (plot c) in comparison to the early Holocene (Fig. 3) where the dynamic term dominates.
[Figure maps created using Python 2.6.6 (www.python.org) on Linux]

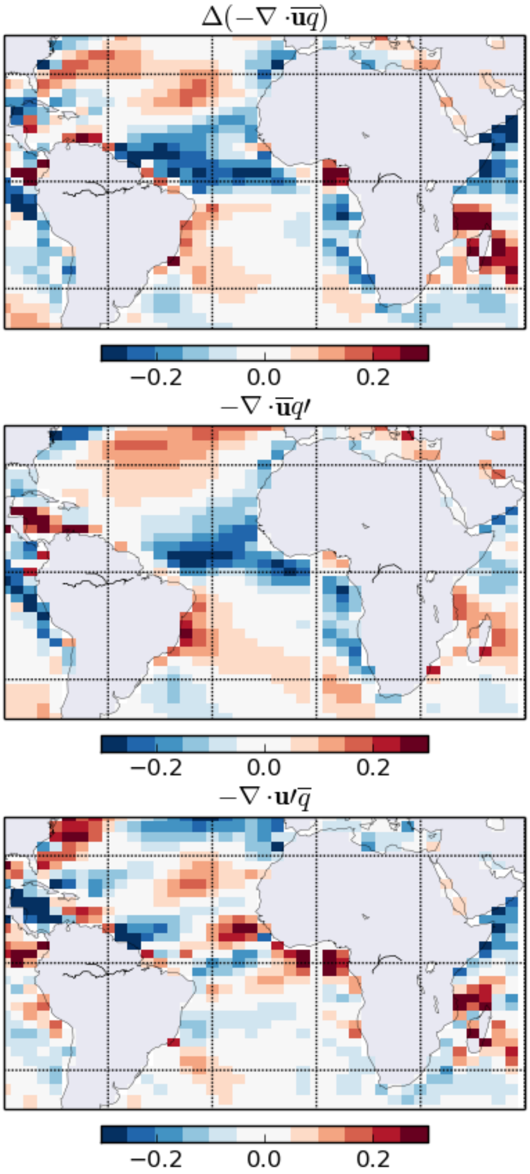

Fig. S8 (a) The Atlantic interhemispheric temperature gradient (320-340°E and 0-15°N vs. 0-15°S) for the ALL experiment in February (blue line), July (red line), and the annual average (black line). (b) Same as (a) but for the latitude of maximum Moist Static Energy (MSE).

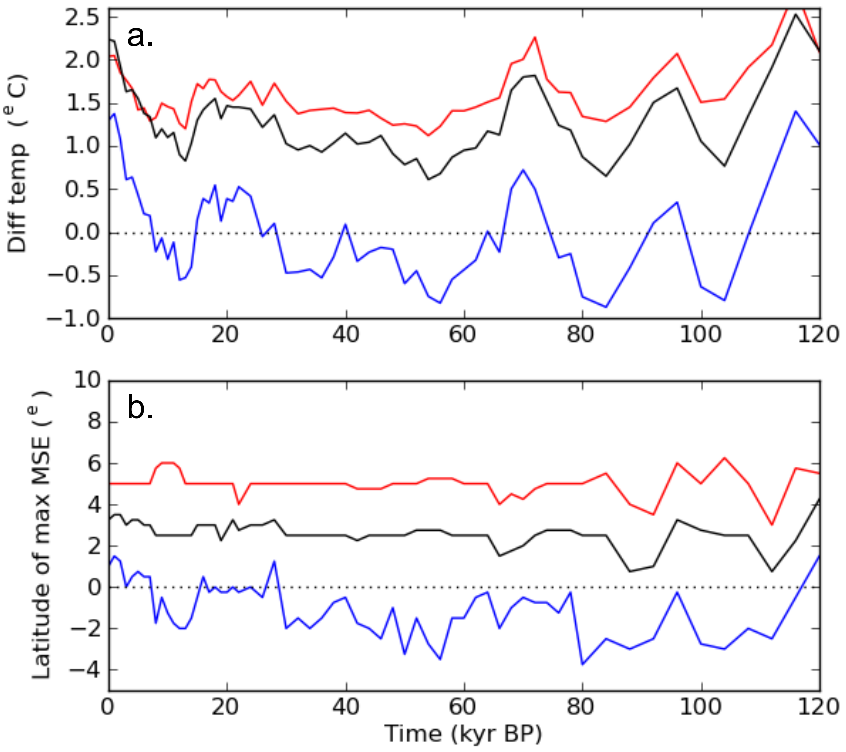

Fig. S9 December-January-February (DJF) mean latitudinal cross-sections from the equatorial Atlantic from the ORB-ONLY (right) and ALL (left) experiments at various time slices, and correlation between various inhemispheric gradients and the DJF tropical rainbelt position. (a) DJF precipitation from 104, 70, 44, and 22 kyr time slices. Square symbols are the calculated southern limits of the rainbelt for DJF for each time slice, and circle symbols are the position of the calculated centroid of the tropical precipitation (i.e. centre of the rainbelt) for comparison. Similar temporal patterns are obtained using both methods of describing the rainbelt (even though the actual latitudes are different). (b) Surface Air Temperature (SAT) from each time slice. (c) Total cloud cover from each time slice. (d) The seasonal cycle of the southern limit of the rainbelt in the Atlantic from the ORB-ONLY (right) and ALL (left) experiments demonstrating the change in seasonality of the 44 kyr time slice when ice-sheet expansion is included. (e) Correlation (using Pearson's correlation coefficient) between various fields and the southern hemisphere rainbelt position from the ORB-ONLY (green) and ALL (blue) experiments. The left two sets of columns correlate the global southern hemisphere rainbelt/ITCZ limit with the global interhemispheric top of the atmosphere radiative budget (IRB) and the global interhemispheric surface temperature gradient (ITG). The two right hand columns correlate the southern Atlantic rainbelt/ITCZ limit with the tropical (0-30°) interhemispheric sea surface temperature gradient and the wider Atlantic region surface temperature gradient (between 50°W and 0°E). The global mean rainbelt southern limit is highly influenced by the Atlantic, and although when only orbital forcing drives the model this leads to a strong influence of the IRB, the non-linear impacts of ice-sheets and their influence on the sensitivity of the rainbelt position to obliquity results in much lower correlation of global IRB/ITG with the ALL southern limit. Only the Atlantic tropical ITG (including local land influence) produces high correlations with the Atlantic rainbelt southern limit for both the ORB-ONLY and ALL simulations.

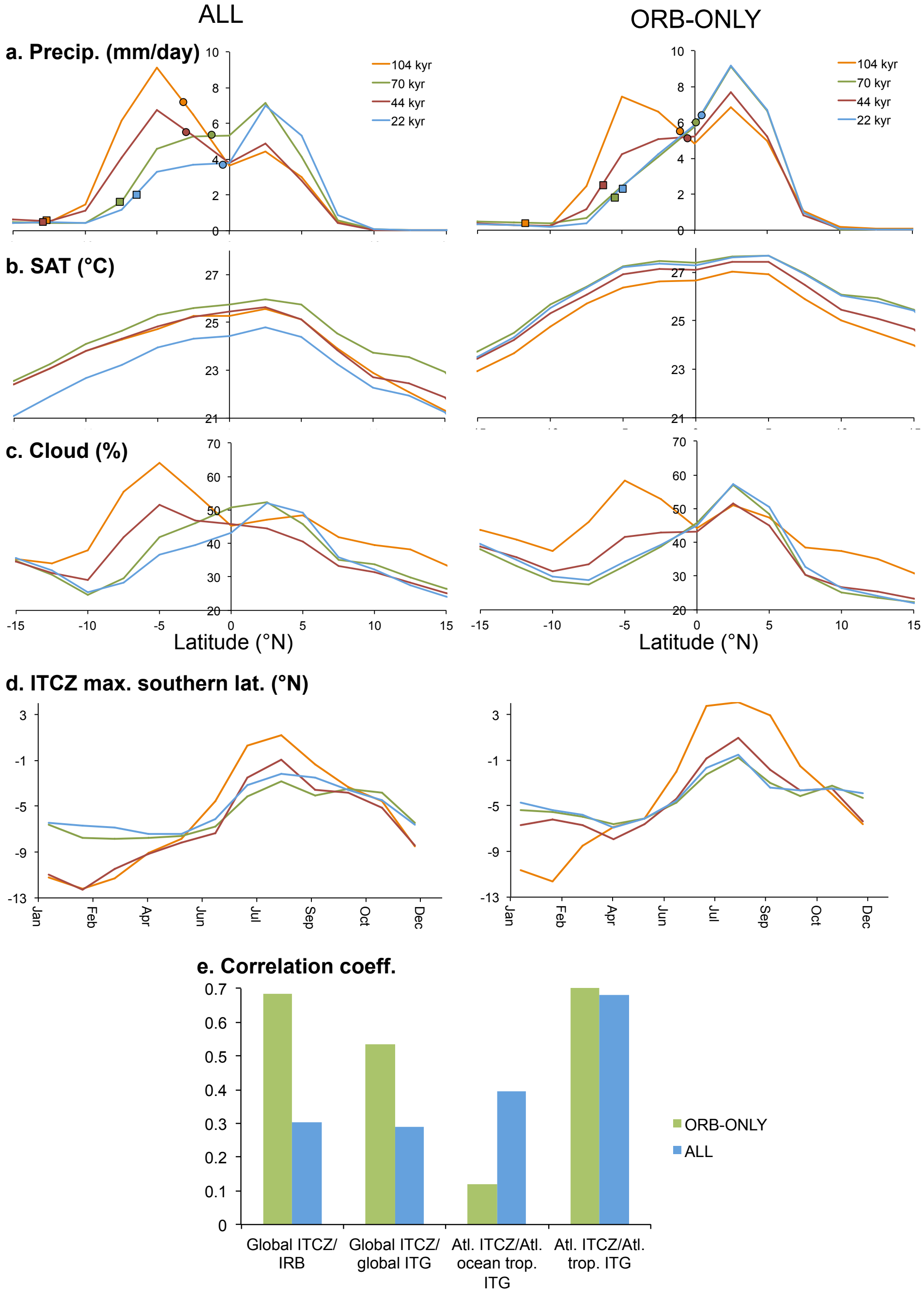

ALL
ORB-ONLY
a. Precip. (mm/day)
104 kyr
70 kyr
44 kyr
22 kyr
b. SAT (°C)
c. Cloud (%)
Latitude (°N)
d. ITCZ max. southern lat. (°N)
Jan
Feb
Apr
Jun
Jul
Sep
Oct
Dec
e. Correlation coeff.
ORB-ONLY
ALL
Global ITCZ/ IRB
Global ITCZ/ global ITG
Atl. ITCZ/Atl. ocean trop. ITG
Atl. ITCZ/Atl. trop. ITG

Fig. S10 Modelled summer (JJA) and winter (DJF) smoothed divergence at 1000 hPa from the ALL experiment: (a) PI, (b) 10kyr – PI. The 10kyr – PI anomalies display sharp zonal shifts in the seasonal signal at the location of the ITCZ, particularly over the Pacific, Atlantic and Africa.
[Created using NCL (https://www.ncl.ucar.edu) version 6.2.1]

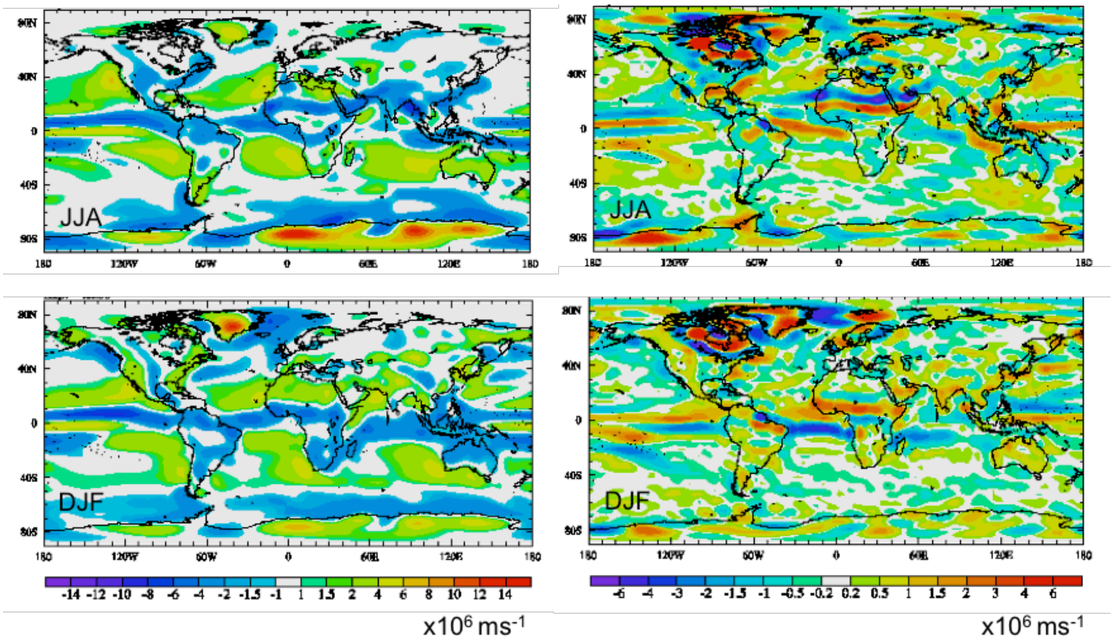

Fig. S11 Top plot shows June, July, August, September (JJAS) average ITCZ latitude (dark blue) at 290-300°W and precipitation (light blue) at 290-300°W and 5-10°N from the ALL experiment to compare to the Cariaco precipitation-proxy record[4]. Bottom plot shows December, January, February (DJF) average ITCZ latitude at 310-320°W and precipitation at 310-320°W and 10°S.

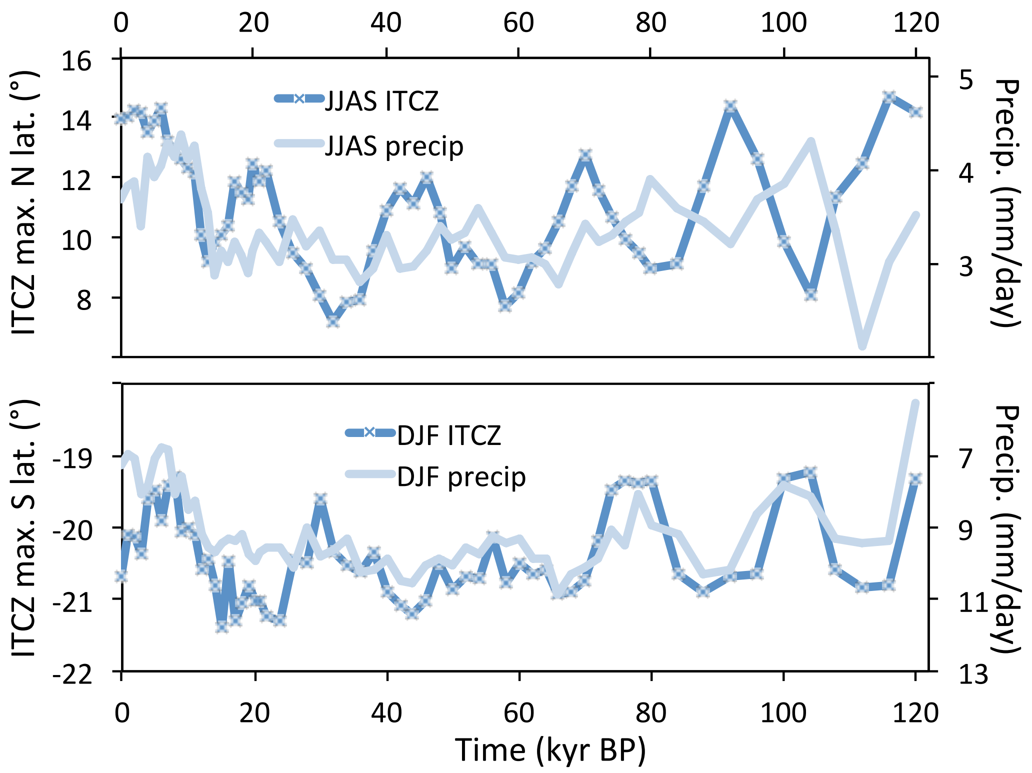

Fig. S12 Orbital configuration parameters through the last glacial cycle. Obliquity (tilt of the Earth to the plane of its orbit), in black, demonstrates high values around 90-95 kyr and 42-48 kyr BP. The precession of the equinoxes (Ecc*sin(prec)) is given in the grey dotted lines.

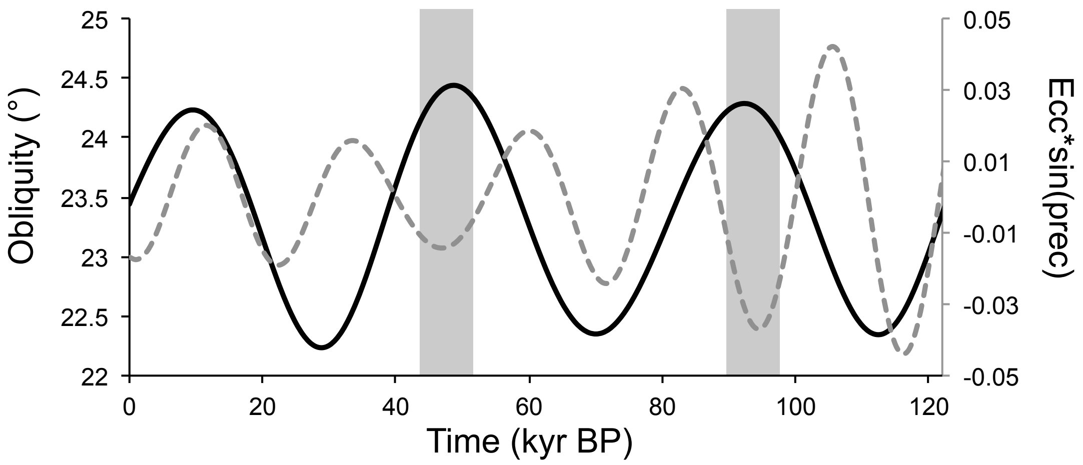

Fig. S13 Monthly mean latitude of the southern Atlantic rainbelt limit over the last glacial cycle for austral summer (January to April) from the (a) ORB-ONLY experiment, and (b) ALL experiment. The comparison shows how at times when the rainbelt is closest to the equator, the most southerly latitude occurs in April, whereas when the rainbelt is farthest south the most southerly extent occurs in February. In the ALL experiment the change in seasonality of the southernmost extension of the rainbelt at times when obliquity is high (~44 kyr and ~ 96 kyr) is clear.

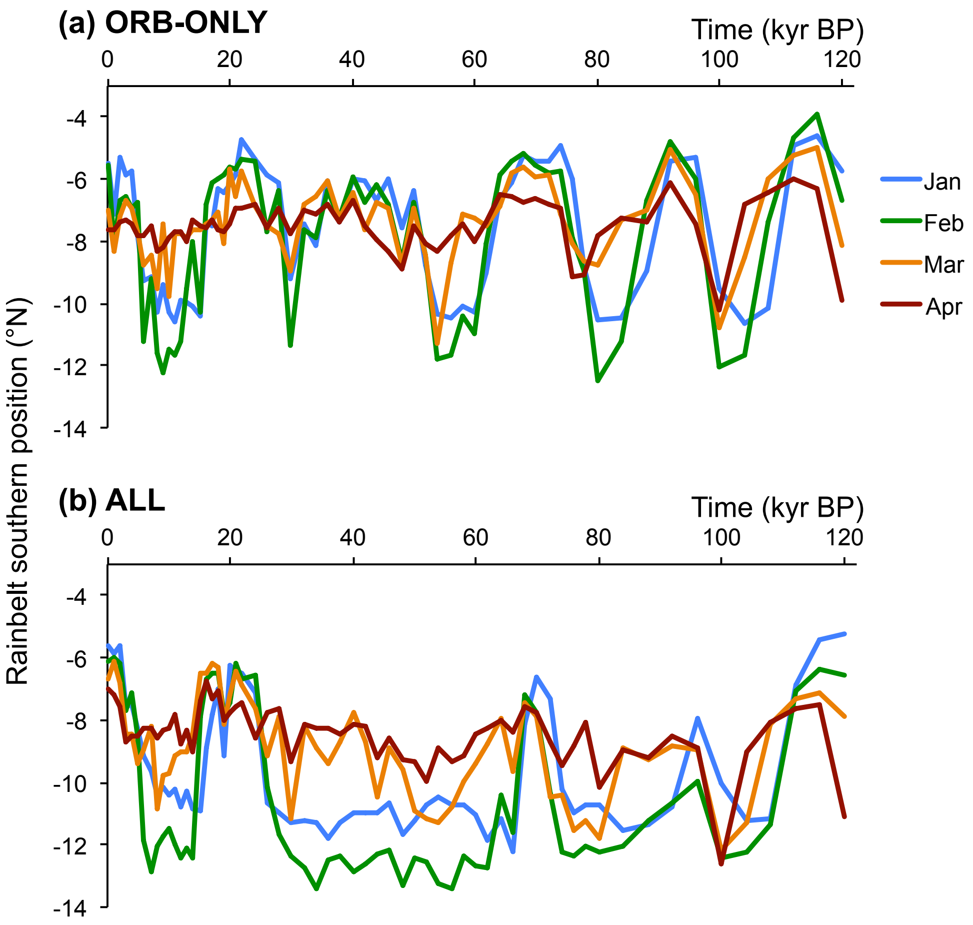

Fig. S14 HadCM3 annual mean precipitation anomalies from pre-industrial (PI) control in the ALL experiment. (a) Mid-Holocene (MH) or 6 kyr BP, and (b) Last Glacial Maximum (LGM) or 21 kyr BP.
[Maps created using Panoply (http://www.giss.nasa.gov/tools/panoply/) version 4.3.1]

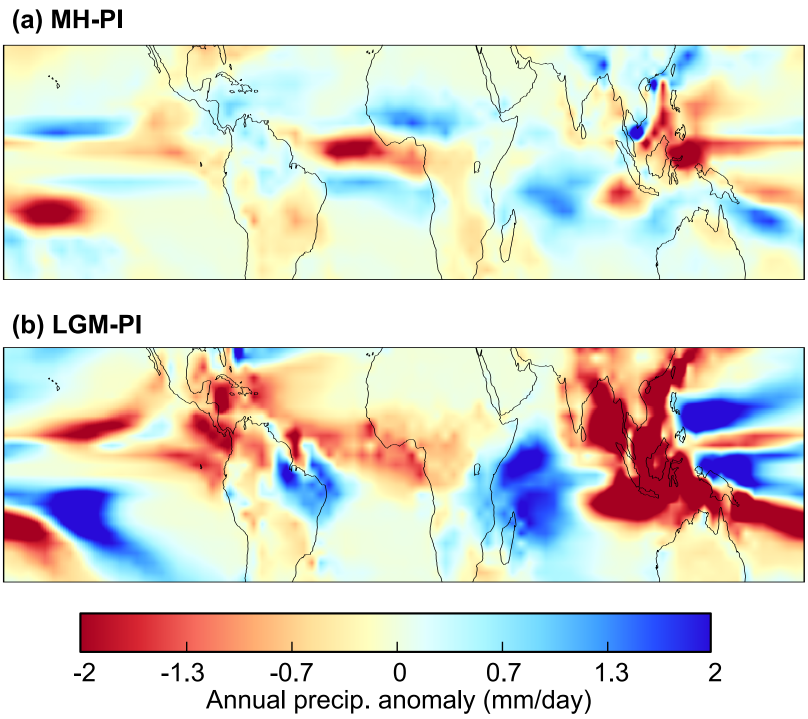

Fig. S15 Last glacial maximum (LGM; 21 kyr BP) minus pre-industrial simulated annual mean precipitation anomalies from a variety of global climate models used in the Paleoclimate Model Intercomparison Project 3 (PMIP3).
[Maps created using Panoply (http://www.giss.nasa.gov/tools/panoply/) version 4.3.1]

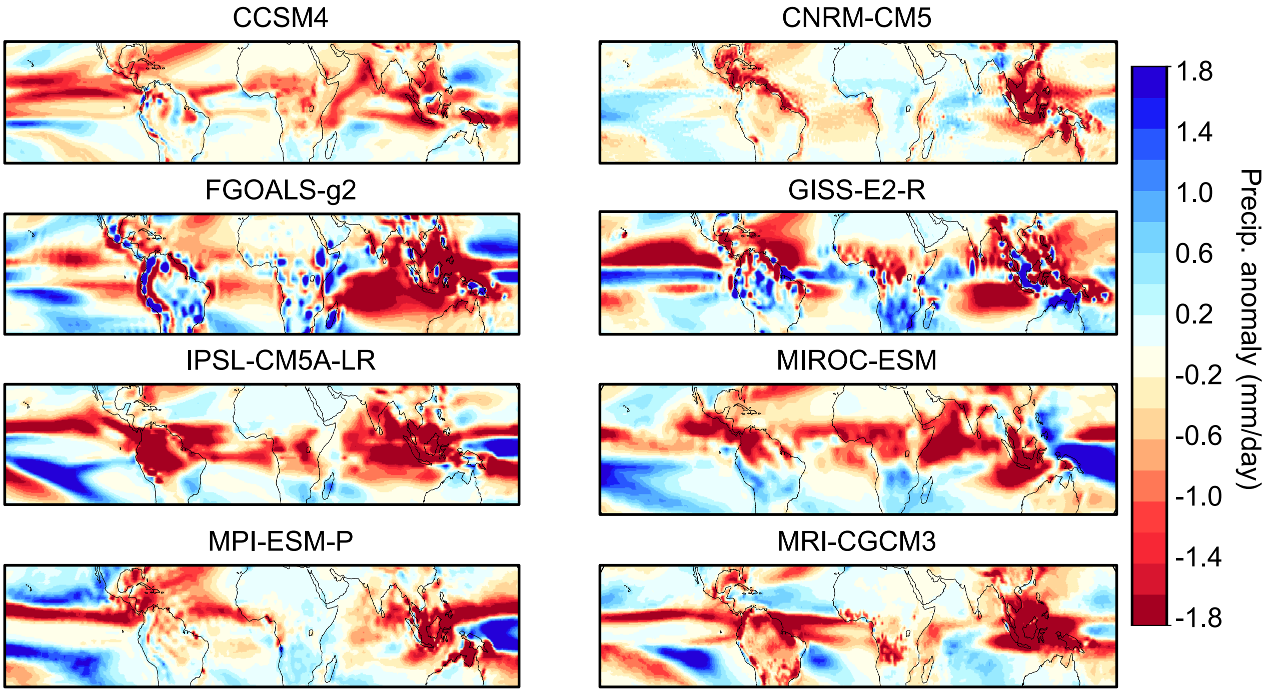

Fig. S16 Latitude of the northern limit of the rainbelt of South America over the last glacial cycle for austral summer months (July to October) from the (a) ORB-ONLY experiment, and (b) ALL experiment.

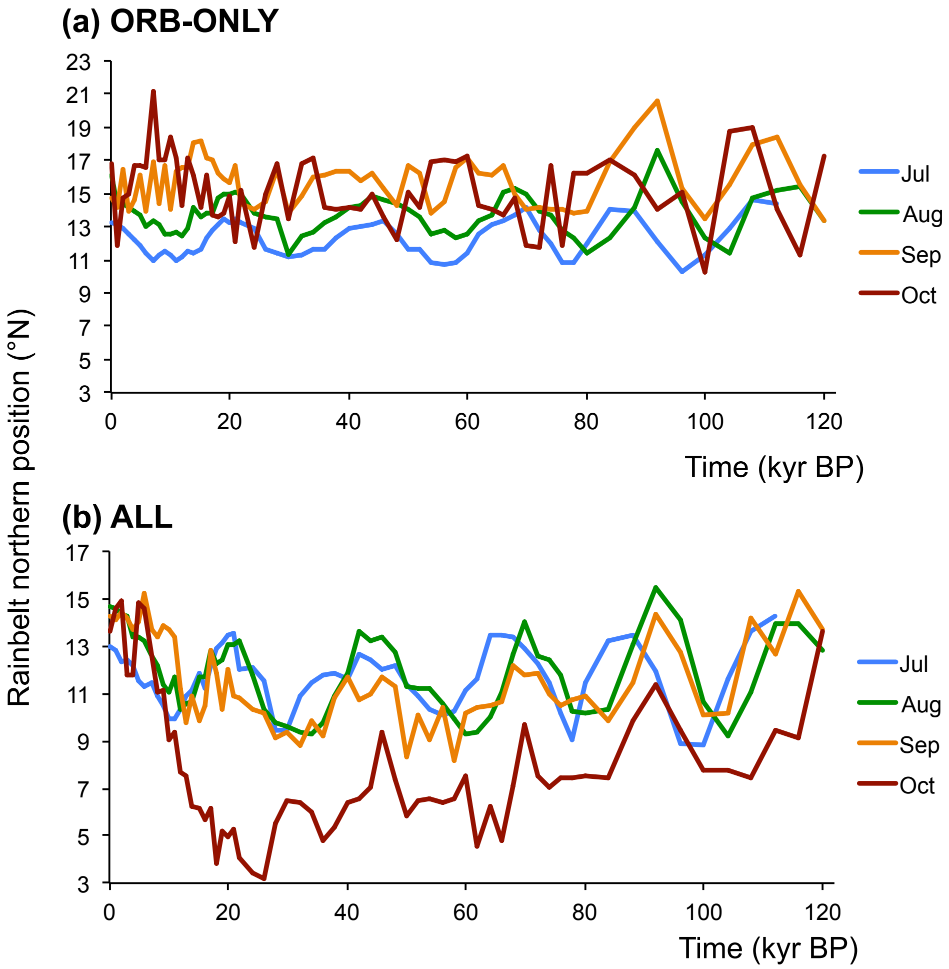

Fig. S17 Same as Fig. 2a, except for the inclusion of uncertainty bars based on the standard deviation of three different 30-year averaging periods for the model simulations at 116kyr (red) and 104kyr (blue).

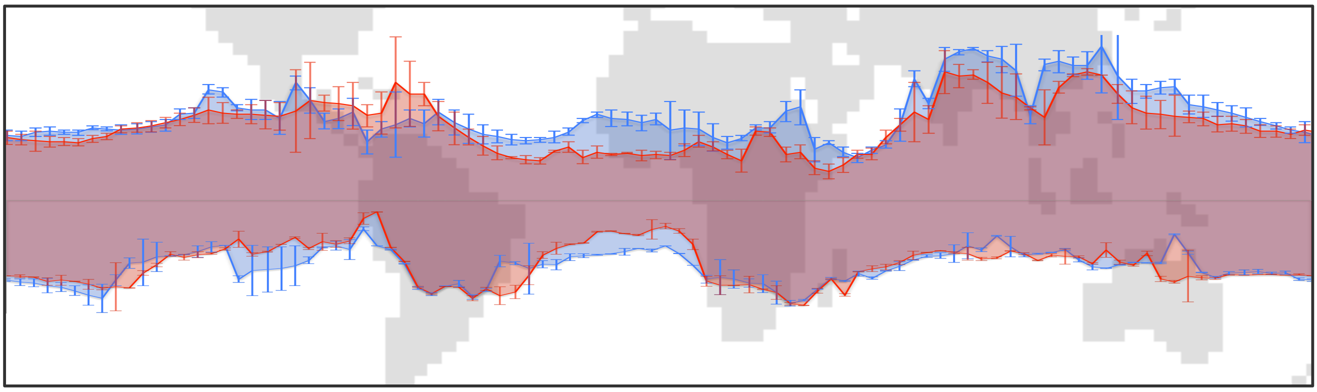

Supplement: Supplementary file 1 — Supplementary Information [file 41598_2017_9816_MOESM1_ESM.pdf]
